# Dietary Intake Contributed the Most to Chlorinated Paraffin Body Burden in a Norwegian Cohort

**DOI:** 10.1021/acs.est.2c04998

**Published:** 2022-11-15

**Authors:** Bo Yuan, Line Småstuen Haug, Joo Hui Tay, Juan Antonio Padilla-Sánchez, Eleni Papadopoulou, Cynthia A. de Wit

**Affiliations:** † Department of Environmental Science, Stockholm University, StockholmSE-10691, Sweden; ‡ Department for Food Safety, Norwegian Institute of Public Health, OsloNO-0213, Norway

**Keywords:** human exposure, cohort study, chlorinated paraffins, plasma, external exposure pathways, dietary intake

## Abstract

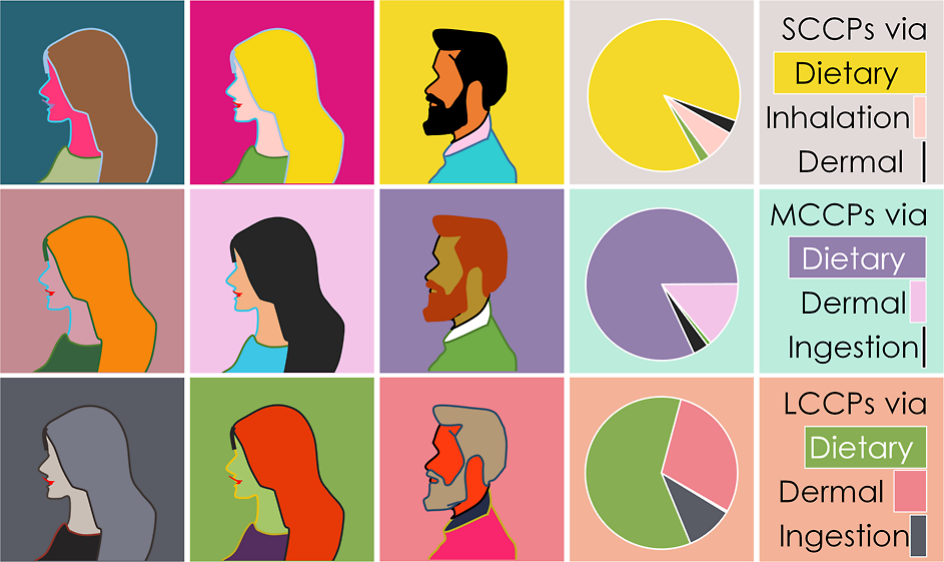

Determining the major human exposure pathways is a prerequisite for the development of effective management strategies for environmental pollutants such as chlorinated paraffins (CPs). As a first step, the internal and external exposure to CPs were quantified for a well-defined human cohort. CPs in participants’ plasma and diet samples were analyzed in the present study, and previous results on paired air, dust, and hand wipe samples were used for the total exposure assessment. Both one compartment pharmacokinetic modeling and forensic fingerprinting indicate that dietary intake contributed the most to body burden of CPs in this cohort, contributing a median of 60–88% of the total daily intakes. The contribution from dust ingestion and dermal exposure was greater for the intake of long-chain CPs (LCCPs) than short-chain CPs (SCCPs), while the contribution from inhalation was greater for the intake of SCCPs than medium-chain CPs (MCCPs) and LCCPs. Significantly higher concentrations of SCCPs and MCCPs were observed in diets containing butter and eggs, respectively (*p* < 0.05). Additionally, other exposure sources were correlated to plasma levels of CPs, including residence construction parameters such as the construction year (*p* < 0.05). This human exposure to CPs is not a local case. From a global perspective, there are major knowledge gaps in biomonitoring and exposure data for CPs from regions other than China and European countries.

## Abstract 2

This is the first time that multiple exposure pathways of all chlorinated paraffin classes are quantified for a single cohort and a major step forward in global exposure assessment of complex mixtures of chlorinated paraffins.

## Introduction

Chemical pollution can cause adverse health impacts on humans and ecosystems at regional or global levels.^1^ The Global Burden of Disease Study 2017 estimated that exposure to 13 chemicals or chemical groups contributed to over 1.3 million premature human deaths across 195 countries.^2^ Wide uses of a chemical increase the chance of human exposure on a daily basis.^3^ For chemicals that are persistent, bioaccumulative, and toxic (PBT), the health impacts can be far-reaching and last for multigenerations.^4^ An indelible lesson learned from the past was the global pollution by the industrial chemical polychlorinated biphenyls (PCBs). People who live far away from the pollution sources have also been exposed to PCBs.^5^ Over 30 years since their near-global ban, PCB-mediated effects on ecosystem health are still significant.^6^

To protect human and ecosystem health from PCBs and other Persistent Organic Pollutants (POPs), the UN Environment Programme adopted the Stockholm Convention in 2001. With the phase-out and banning of PCBs, their uses have been substituted by closely related chemicals,^7^ among which chlorinated paraffins (CPs) are primarily used.^8^ Nowadays, CPs have become one of the most popular plasticizers, additives to metal-machining fluids, paints, coatings and sealants, and flame retardants.^9^ The annual global production of CPs, over 1.3 million metric tons, exceeds the total amount of PCBs that have ever been produced.^10^ CPs are synthesized by chlorination of straight alkanes. Poor positional selectivity in the production leads to CP products that are complex mixtures of C_*n*_H_2*n*+2-*m*_Cl_*m*_ isomers.^11^ Based on the number (*n*) of carbons, CP products are classified into short-, medium, and long-chain CPs (SCCPs, MCCPs, and LCCPs) for *n* = 10–13, 14–17, and >17, respectively. Some CP products may also contain impurities of very-short chain CPs(vSCCPs) with *n* < 10.^12^ CP isomers that have the same numbers of carbons and chlorines are denoted as a CP homologues (C_*n*_Cl_*m*_). There are theoretically 600–700 CP homologues, and the homologue profile has been used as an environmental forensic fingerprint.^13^ However, in most cases, CPs are produced, studied, and regulated on the basis of S/M/LCCP mixtures.

CPs are global contaminants.^14^ Very recently, they have been found in human mothers’ milk from over 50 countries across five continents and accounted for 18–46% of the total POPs concentration in the samples.^15^ Similar to many POPs, all the CPs are persistent and bioaccumulative.^16,17^ Developmental toxicity was shown to be a sensitive endpoint for the mammalian toxicity of CPs,^18^ while endocrine disruption effects have been found for different CP classes.^19^ Theoretically, the adverse effects of CPs could further increase during metabolic transformation in human body.^20^ The environmental evidence of PBT properties is available mostly for SCCPs, which became the first CP class to be listed for global restriction under the Stockholm Convention for POPs in 2017. As a consequence, the high-volume production of CPs is now tilted in favor of the current-use CPs, that is, MCCPs and LCCPs, as alternatives to the legacy SCCPs.^21^ This may increase the emissions and possibly the exposure to MCCPs and LCCPs. Regulatory actions have been taken to evaluate MCCPs^22^ and LCCPs as POPs.^23^ For developing effective management strategies of these chemicals, it is crucial to comprehensively determine major exposure pathways for humans.^24^

**Figure 1:**
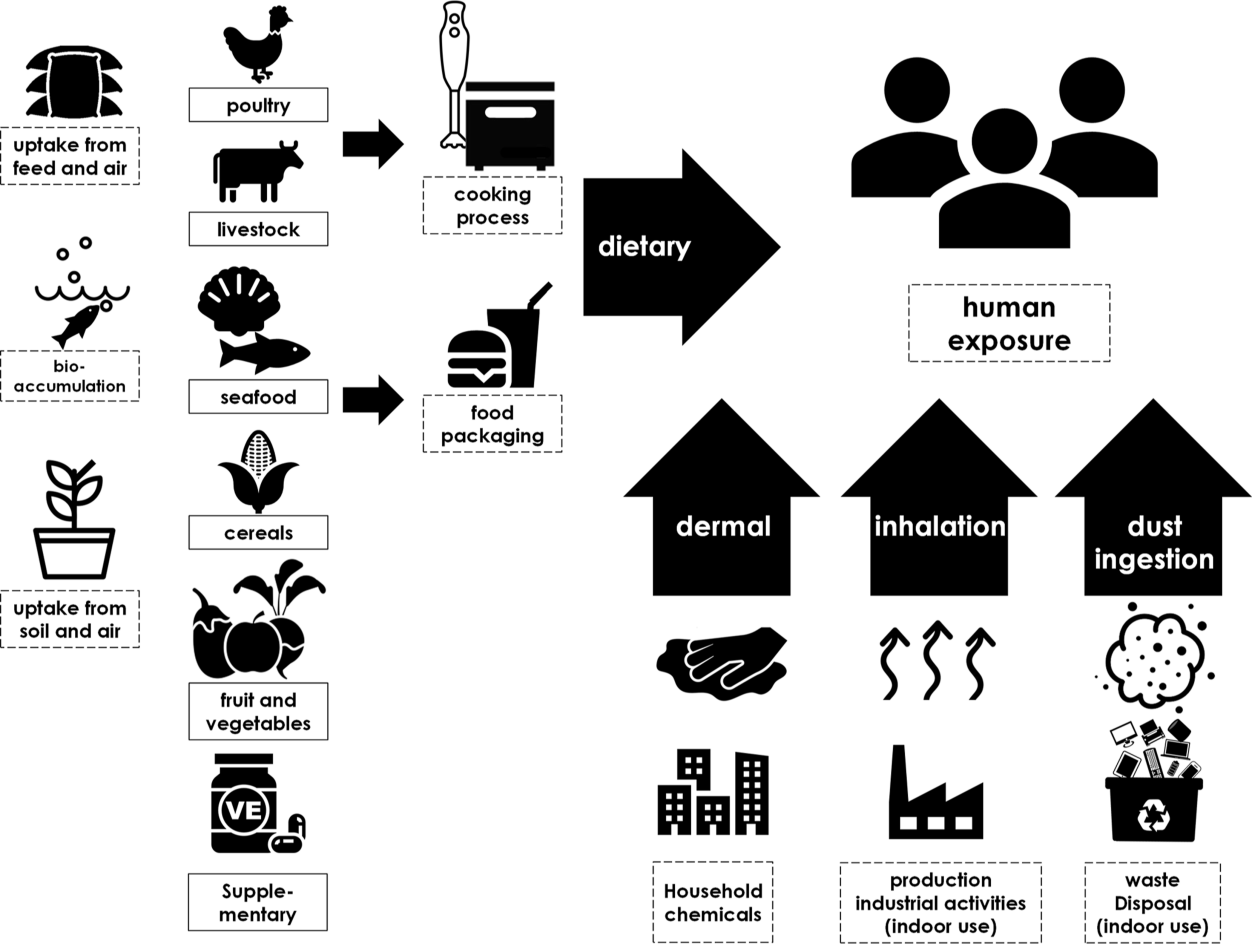
Overview of possible CP exposure pathways to the human population. For detailed references to individual pathways, see Table S1.

The ubiquity of CPs in the environment contributes to diverse exposure pathways. An overview of transport and fate of CPs that can result in human exposure is summarized in Figure 1. Four exposure pathways have become evident based on previous exposure assessments, which are dietary,^25,26^ inhalation,^27^ dust ingestion,^28^ and dermal exposure.^29,30^ Correlations between the external exposure and human internal levels of CPs have rarely been studied. Blood levels of CPs in a general population were estimated based on local diet, air, and soil data by Dong et al.^31^ using a physiologically based pharmacokinetic (PBPK) model in rats. However, the monitoring data, at present, are lacking to corroborate estimated body burden from external exposures with actual internal exposure measurements in the same individuals.

Our previous works assessed human exposures to all CP classes via inhalation, dust ingestion, and dermal exposure for a well-defined human cohort,^27,30^ comprising 61 participants recruited in Norway in 2013–14.^32^ To quantify the internal exposure and the total external exposures to CPs, the current study complimented the previous work by (1) assessing exposure from dietary intake of CPs by analyzing pooled diet samples collected on two consecutive days and (2) calculating individual participant body burdens of CPs based on analysis of plasma CP concentrations. Together with the previous results of inhalation, dust ingestion, and dermal exposure,^27,30^ we explore the relative contributions of multiple exposure pathways to the body burdens of CPs using a one compartment pharmacokinetic (PK) model and a forensic fingerprinting approach. This study, as the first cohort study on CPs, contributes considerably to the exposure assessment of complex mixtures of CPs.

## Methods and Materials

### Cohort Study

The study recruited 61 adult participants from the staff of the Norwegian Institute of Public Health, Oslo, Norway, consisting of 45 women and 16 men with ages ranging from 20 to 66.^32^ There was no known occupational exposure to CPs for the participants. The participants duplicated the portions of all foods consumed over two consecutive days between November 2013 and May 2014. The duplicated diet of each day was collected in a precleaned polypropylene (PP) bottle as one subsample. Total mass of food as well as types of food and packaging material were recorded in a food record. During the same sampling period, the stationary air and settled dust from the resident’s living room and hand wipe samples were collected, in which CPs have been analyzed in our previous studies.^27,30^ A venous blood sample was collected from each participant, a portion of which was centrifuged for plasma for CP analysis.^32^ The blood samples were collected before or after the 2-day sampling period, at the convenience of the participants. In addition, the participants filled out a food frequency questionnaire, a food diary, and a questionnaire on housing characteristics. For more details, see the Supporting Information under Text S1 Sample Collection and Table S2.

This study was approved by the Regional Committees for Medical and Health Research Ethics in Norway (Case number 2013/1269), and all participants completed a written informed consent form prior to participation. Approval for the chemical analyses carried out in Sweden was given by the Regional Ethics Committee in Stockholm, Sweden (Case number 2014/624–31/1). The results and data of the current study are presented in a form fulfilling the requirements of the informed consent and the European General Data Protection Regulation (GDPR).

### Sample Preparation

Plasma samples from 59 out of the 61 participants were available for this study. The total lipid extraction method was adopted from Sahlström et al. 10 ng of internal standard ^13^C_10_-1,5,5,6,6,10-hexachlorodecane (^13^C_10_-HCD, Cambridge Isotope Laboratories, Andover, MA) was added to 1–2 g plasma sample. The samples were liquid–liquid extracted with a mixture of 2-propanol, *n*-hexane, and methyl *tert*-butyl ether. The extract was then cleaned with a solution of potassium chloride. The aqueous phase was re-extracted with *n*-hexane, and the combined organic phases were gently evaporated to dryness. The lipid weight was determined gravimetrically.

The diet samples from 2 out of the 61 participants had been used up in the previous tasks of the cohort study. Therefore, diet samples from 59 participants were available for the current study. A duplicate portion of all foods ingested over 24 h was collected for 2 consecutive days. Based on the food weight of each day, the diet samples of the 2 days were mixed proportionally into one diet sample for each participant. The diet samples were extracted according to Yuan et al.^13^ The mixed diet samples were freeze-dried, and water contents were determined gravimetrically after freeze-drying. A 2–3 g sample was spiked with ^13^C_10_-HCD and extracted using accelerated solvent extraction (ASE 300; Dionex Europe, Leeds, UK). The extract was gently dried, and lipid content was determined gravimetrically.

The lipid extracts of plasma or diet samples were cleaned-up on a multilayer SPE column.^33^ The eluent was reconstituted in dichloromethane (DCM) with 20 ng of Dechlorane-603 (Occidental Chemical Corp.) prior to instrumental analysis. For more details, see the Supporting Information under Text S2 Sample Preparation.

### Chemical Analysis

A total of 675 CP homologues (C_*n*_Cl_*m*_, where *n* > 5, *m* > 1) were analyzed using a chloride-enhanced UPLC-APCI-Orbitrap-HRMS (Q Exactive, Thermo Fisher Scientific, San Jose, USA). The relative abundances of the 675 homologues of a sample made up a C_*n*_Cl_*m*_-profile. SCCPs, MCCPs, and LCCPs in the samples were quantified using a C_*n*_Cl_*m*_-profile deconvolution method^34^ with 9 SCCP, 6 MCCP, and 5 LCCP reference standards, respectively (Table S3). The concentrations of vSCCPs were quantified based on a Chinese CP mixture (CP-52).^12^ For detailed instrumental parameters^27^ and the quantification methods,^17^ see Text S3.

### Dietary Exposure Calculation

For each participant, the daily intake of CPs via food [ng/kg Body Weight (BW)/d] was calculated according to eq 1:^35^
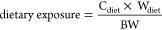
1where C_diet_ is the wet weight-based concentration of vSCCPs, SCCPs, MCCPs, or LCCPs in the diet sample (ng/g ww), W_diet_ is the average of the total food consumption (g/day) for the two consecutive days, and BW is the participant’s body weight (kg).

### Estimation of Plasma Concentrations from Intake Data

The concentration of CPs in the lipids (ng/g lipid) was calculated using a simple one-compartment, first-order PK model^36^ based on the calculated intakes from the four external exposure pathways. At the steady state, the term ∑(exposure_CPs_×AF_CPs_) is the total daily exposure (ng/kg BW/d) to CPs for the Norwegian cohort calculated as the sum of exposure through diet (the current study), inhalation, dust ingestion,^27^ and dermal intakes,^30^ the calculation of which has included absorption fraction (AF) or bioaccessibility (Table S4): 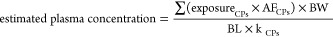
2The relative contribution of each exposure pathway is calculated based on its share of the total daily exposure (Text S4). BL is the body lipid mass estimated from each individual participant’s height and body weight (g) (Text S5). k_CPs_ is the compound-specific first-order dissipation rate (day^–1^) and calculated as 0.693/*t*_0.5_, where *t*_0.5_ is the halflife of CPs^31^ in the body lipid compartment (Table S4). Due to the low detection frequencies in multiple matrices, vSCCPs were not included in the estimation.

### Forensic Fingerprinting

CPs in each sample are fingerprinted in the form of C_*n*_Cl_*m*_-profile, which has been used for indicating contamination source(s).^13^ The sample can show a fingerprint similar to that of the potential source. When there were more than one potential sources of CPs, the sample fingerprint can be deconvolved into a superimposition of several source fingerprints, and relative contributions of each source can be assessed using this approach. The approach was adopted from a previous study^13^ with additional consideration of bioaccessibility of CP homologues. The C_*n*_Cl_*m*_-profile of each plasma sample was linearly superimposed using the bioaccessibility-calibrated profiles of the corresponding four external exposure media. Relative contributions of individual exposure pathways to the internal CPs were calculated. The superimposition was optimized for each participant with the goal of maximizing the goodness-of-fit R^2^ between the plasma profile and the superimposed one. *R*^2^ ranges were between 0 and 1, and *R*^2^ = 1 means a perfect superimposition. More details and examples can be found in the Text S6 and Figure S1.

### QA/QC

A spike-and-recovery test was performed for serum samples. For details, see Text S7. The method validation for dietary samples can be found in a previous study.^13^ The recoveries of ^13^C-labeled CP internal standard (mean ± SD) were 99 ± 23% and 76 ± 16% for diet and plasma results, respectively. The resolution of MS (120,000 full width at half maximum) is considered capable of resolving CPs at C_*n*_Cl_*m*_ levels.^37^ The quantification performance was evaluated with R^2^ between the measured C_*n*_Cl_*m*_ profile and the deconvolved one. When *R*^2^ < 0.5, the results were reported as tentative values. Quantification of vS/S/M/LCCPs of all the samples fulfilled the criterion of *R*^2^ ≥ 0.50. Sampling tools, containers, and glassware were prewashed and rinsed using ultrapure solvents. Glassware was heated in a furnace at 450 °C overnight before use. As field blanks for the diet samples, methanol was added to the same type of bottles as those used for the samples. The field blanks were concentrated and transferred in LC vials for instrumental analysis. A laboratory blank was included in every batch of samples to assess background contamination of CPs during sample treatment. The method detection limit (MDL) was defined as mean laboratory blank value plus three times the standard deviation. The MDLs of vSCCPs, SCCPs, MCCPs, and LCCPs were 0.27, 1.33, 2.86, and 0.32 ng/g ww for diet samples, respectively, and 43, 510, 590, and 51 ng/g lipid for plasma samples, respectively. CPs in the field blanks were below the MDLs.

### Statistical Analysis

Concentrations below the MDL were replaced with MDL/√2.^38^ Pearson’s correlation analysis was performed among different sample media, and the CP concentrations were log-transformed prior to the testing. The distributions of CPs in both the plasma and the diet samples were highly skewed. Therefore, the Mann–Whitney U and Kruskal–Wallis tests were used to explore differences between CP amounts in samples (with >75% detection frequency) and categorical variables such as food types, living habits, and residential environment documented in the questionnaires/diaries, while the Spearman’s rank correlation was used for continuous variables. PAST (Version 4.10)^39^ was used for statistical analysis. The level of significance was set to *p* = 0.05.

## Results and Discussion

### CPs in the Diet

Over 98% of the diet samples in the cohort had SCCPs/MCCPs above the MDLs, and the total concentrations of CPs ranged between 4.5 and 55 ng/g ww (Table 1). MCCPs were the most abundant CP class, which contributed a median of 61% of the total CPs, followed by SCCPs and LCCPs, which contributed 28 and 5.8%, respectively.

**Table 1:** Descriptive Statistics for CPs Measured in Diet Samples (*n* = 59) and Estimated Daily Dietary Exposure to CPs for the Participants

CP category	vSCCPs	SCCPs	MCCPs	LCCPs	sumCP
DF	58%	98%	98%	92%	
concentration (ng/g ww)					
geometric mean (GM)	<0.27	5.6	13	1.2	20
median	0.39	6.4	12	1.1	23
range	<0.27–3.0	<1.3–16	<2.9–35	<0.32–14	4.5–55
chlorine content (w/w)					
geometric mean	62% Cl	59% Cl	52% Cl	48% Cl	54% Cl
estimated daily dietary exposure (ng/kg BW/d)					
5th Percentile	N.A.	14	36	2.3	54
median	2.9	42	96	8.5	160
95th Percentile	8.8	120	250	38	400
RfD^a^	N.A.	2 300	6 000	71 000	N.A.

a The sum of CPs (sumCP) was calculated from individual results. The detection frequency (DF) is the percentage of samples with a concentration above the MDLs; reference dose (RfD) values for SCCPs, MCCPs, and LCCPs were calculated by dividing respective LOEL values (2.3,^40^ 6, and 71^41^ mg/kg bw/d) by a safety factor of 1000;^18^ N.A.: not available.

The estimated median dietary intake of total CPs for the participants in the cohort was 160 ng/kg BW/d (Table 1). Human adult exposure to CPs via diet worldwide is summarized in Table S5. The dietary exposure to SCCPs and MCCPs in the Norwegian cohort (GM: 42 and 96 ng/kg BW/d, respectively) was in the medium range of levels among the European countries such as Germany (mean 63 and 54 ng/kg BW/d, respectively)^26^ and France (mean 135 and 175 ng/kg BW/d, respectively)^42^ and was about one order of magnitude lower than that in China (mean 260–1300 and 180–940 ng/kg BW/d, respectively)^31,43^ and Korea (mean 781–888 ng SCCPs/kg BW/d).^44^ The estimated 95th percentile dietary intake of CPs in the present study was at least one order of magnitude below the respective reference doses (RfDs).

### CPs in Plasma Samples

Up to 21000 ng/g lipid of CPs was found in the plasma samples from the participants (Table 2). SCCPs were the most abundant CPs, which contributed a median of 66% of the total CPs. MCCPs and LCCPs contributed on a median of 29 and 3.2%, respectively, of the total CP concentrations. The median plasma level of legacy SCCPs in this cohort (2500 ng/g lipid) was lower than levels reported in China (3500 ng/g lipid^45^ and 16100 ng/g lipid^46^), while the median levels of the current-use MCCPs (1100 ng/g lipid) and LCCPs (120 ng/g lipid) were comparable to those in China (740^45^–1340^46^ ng/g lipid and 150 ng/g lipid,^45^ respectively, Table S6).

**Table 2:** Descriptive Statistics for Measured CP Concentrations in Plasma Samples (*n* = 59) and the Ratios Between Individual Predicted and Measured Concentrations

CP category	vSCCPs	SCCPs	MCCPs	LCCPs	sumCP
DF	58%	86%	76%	75%	
measured concentration (ng/g lipid)					
geometric mean	51	2100	1300	110	3700
median	68	2500	1100	120	4200
mean	96	3100	2200	180	5600
range	<43–350	<510–10000	<590–9800	<51–700	<<MDL-21000
chlorine content (w/w)					
geometric mean	61% Cl	57% Cl	51% Cl	45% Cl	54% Cl
the ratio between predicted concentration and measured concentration					
median	–a	1.0	0.48	0.15	0.78
mean	–a	1.5	0.70	0.67	1.2

a vSCCPs were not calculated due to a low detection frequency.

### Associations Between Human Biomonitoring Data and External Exposure Data

The relationships of CPs in the four external media with the corresponding levels in the plasma were assessed (Table S7). Positive and significant correlations were seen between concentrations of SCCPs, MCCPs, and LCCPs in the plasma and diet. No significant correlations were found between CPs in plasma and the other exposure media. Personal air samples were available for 13 participants in the cohort. SCCP and MCCP concentrations in the personal air samples showed higher Pearson’s correlation coefficients (*r*) with the corresponding plasma samples than the paired stationary air samples (Table S8). However, these results need to be interpreted with caution, given the small sample sizes.

**Figure 2:**
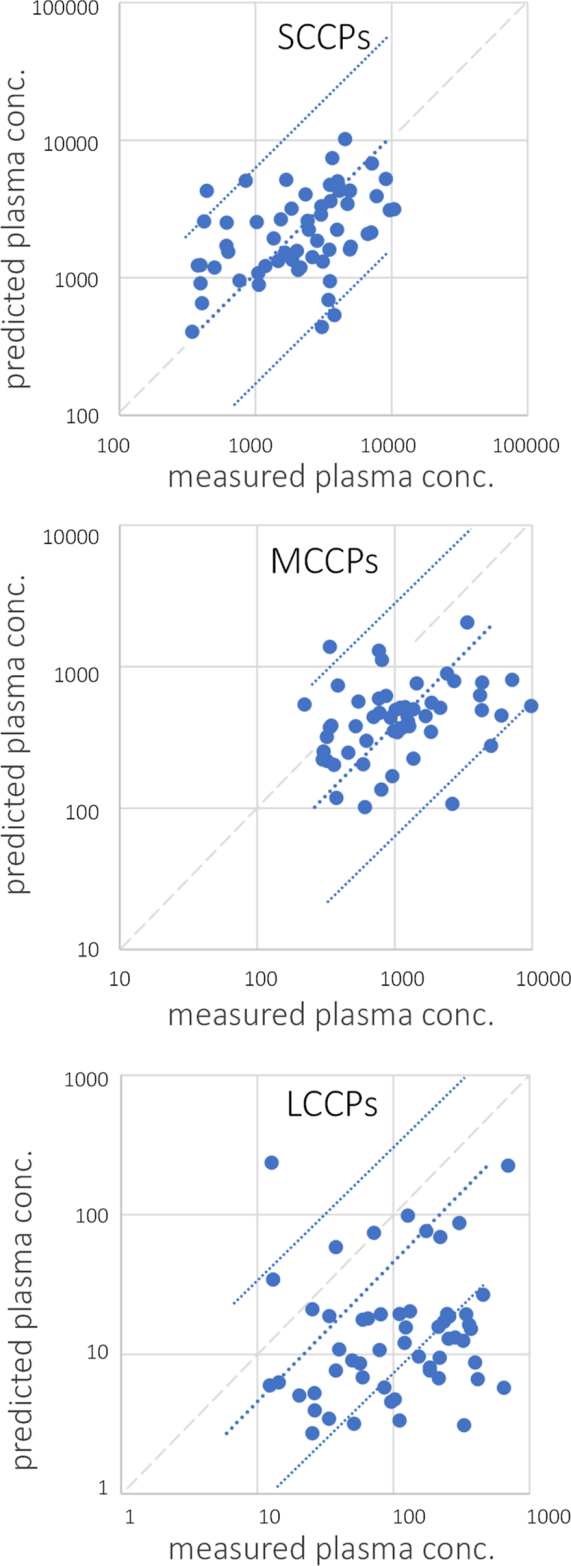
| Measured versus predicted plasma concentrations of SCCPs, MCCPs, and LCCPs. Both concentrations (ng/g lipid) were plotted using log-transformed data. The intercepts were set to zero. The dotted blue lines show the 95% confidence intervals of the predicted mean. Gray dashes show the linear correlation of *x* = *y*. For tabular summaries, see Table S9.

The plasma concentrations of CPs were predicted using the PK model, and the predicted concentrations were compared to the measured concentrations in Figure 2. The plasma concentrations of SCCPs were predicted well by the model based on the external exposure data, with a slope of 1.08 (*r* = 0.77). The plasma concentrations of MCCPs and LCCPs were underestimated by a median factor of 2.1 (mean factor 1.4, *r* = 0.63) and 6.7 (mean factor 1.5, *r* = 0.50), respectively (Table 2). The deviation is comparable or lower than those reported for other halogenated flame retardants such as BDE-47 (a factor of 5.5) and BDE-209 (a factor of 13) in the same cohort,^47^ and for BDE-209 (a factor of 14)^36^ using samples that were not paired. In a study using the PBPK model, the average blood concentrations of SCCPs, MCCPs, and LCCPs on a wet weight basis were predicted for two Chinese cities, which were a factor of 0.86–1.06, 1.01–1.28, and 1.02, respectively, of the measured concentrations.^31^ Unlike SCCPs, MCCPs and LCCPs are currently in use in the European countries. Given the diverse uses of CPs, it is likely that not all exposure pathways were identified and included in the prediction model for MCCPs and LCCPs, which resulted in an underestimation of the plasma concentrations. The relatively large underestimation of LCCP plasma concentrations in the current study could also be due to high uncertainties in the estimated absorption fraction of LCCPs and/or the estimated half-life (Table S4). In addition, the steady-state condition is assumed using the PK model, which is an inherent uncertainty with the approach.^47^

### Relative Contribution of Each Exposure Pathway

On a population level, dietary intake was the predominant exposure route to SCCPs, MCCPs, and LCCPs in the cohort, contributing a median of 88%, 82%, and 60% of the total daily intakes, respectively (Table S9). Inhalation was the second most important exposure pathway for SCCPs (median 6.5%), while its relative contribution to the total CP intake decreased in the case of MCCPs (0.90%) and LCCPs (0.38%), which may be due to a decrease in the volatility with increasing chain length. On the contrary, the relative contributions of dust ingestion and dermal exposure increased from SCCPs to LCCPs. Dermal intake contributed a median of 29% of the total LCCP intake, while dust ingestion contributed 10%. These trends might be due to the increased molecular weight and hydrophobicity going from SCCPs to LCCPs (Table S4).

**Figure 3:**
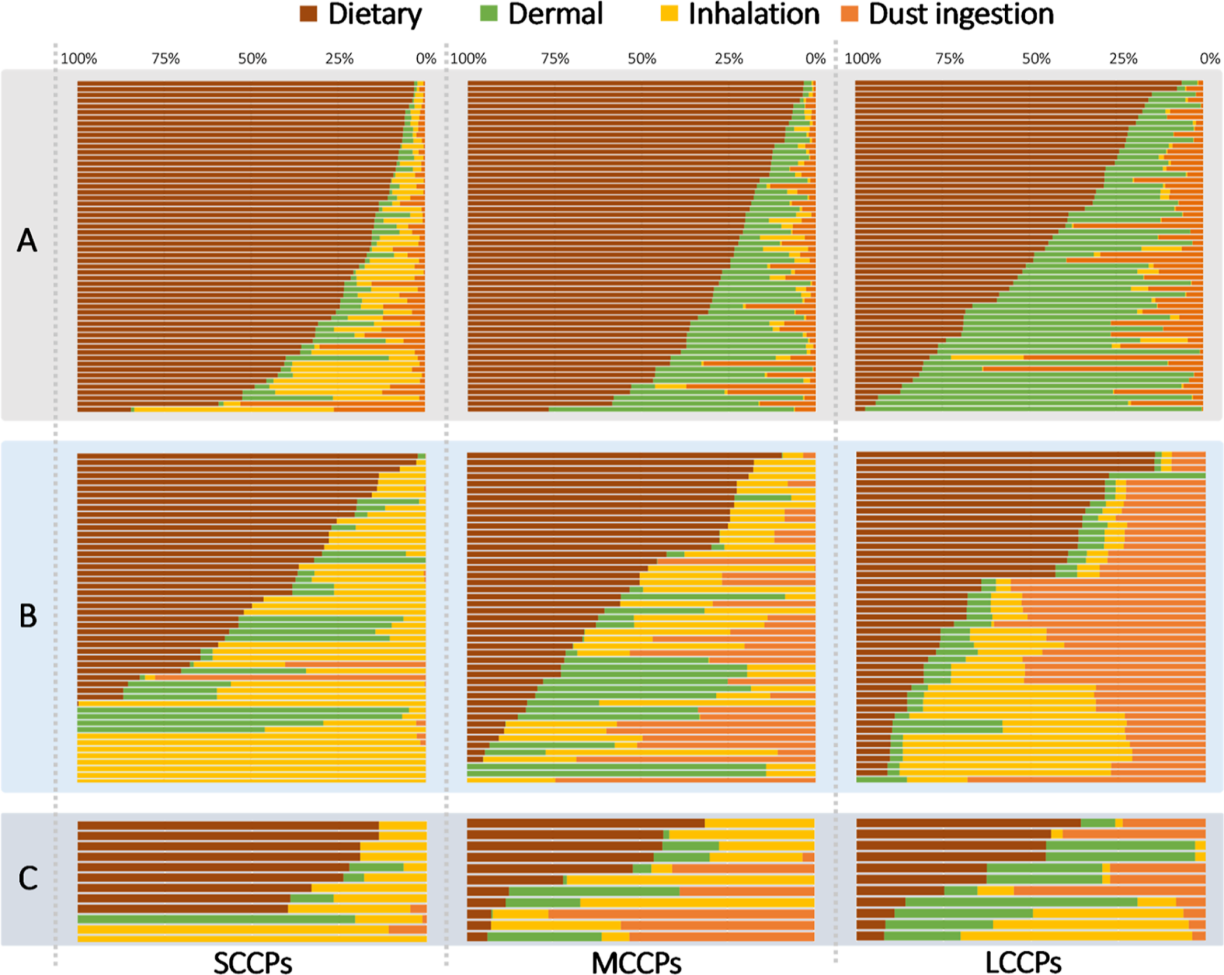
| Relative contributions of different external exposure pathways for SCCPs, MCCPs, and LCCPs for individual participants. (A) Calculation based on the estimated intakes from individual external exposure pathways; (B) reconstructed plasma results using the forensic fingerprinting approach using stationary air data; (C) results using the forensic fingerprinting approach using personal air data. For tabular summaries, see Table S9.

One advantage of a cohort study like this is that it contains personal exposure information, and thus individual variation can be studied. Therefore, relative contributions of external exposure pathways were further explored on the basis of individual participants (Figure 3A). As can be seen in Figure 3A, the importance of the different external exposure pathways showed large variations on an individual basis. We compared the external exposure pathways between the 10 participants with the highest plasma concentrations (further referred to as top 10 group) with the rest of the cohort (Table S10). The median dietary intake of SCCPs, MCCPs, and LCCPs was 1.6-, 1.5-, and 1.2-times higher, respectively, in the top 10 group than in the rest, but the difference was not statistically significant (*p* > 0.05). Nevertheless, the contribution from dietary intake to the total exposure was significantly higher in the top 10 group (*p* < 0.05). Comparisons were also made between the 10 participants with the lowest plasma concentrations (further referred to as bottom 10 group) and the rest of the cohort. Significantly lower dietary intakes of LCCPs were found in the bottom 10 group (median: 0.3 ng/kg BW/d) than the rest (median: 0.6 ng/kg BW/d).

### Forensic Fingerprinting

The average C_*n*_Cl_*m*_ homologue profiles of the diet and plasma samples are shown in Fig. S2. The C_*n*_Cl_*m*_ fingerprint of each plasma sample was reconstructed with the fingerprints of the four external pathway samples, and the relative contributions of external pathways were plotted for individual participants in Figure 3B. The forensic fingerprinting results confirmed the main findings using the intake estimation. CPs in the diet contributed most to exposure, the contributions of CPs from inhalation decreased from SCCPs to LCCPs, while the contribution of CPs from dust ingestion and dermal exposure were higher for LCCPs than for SCCPs (Table S9).

The relative contributions of CP profiles from dietary intake to the corresponding plasma profiles (median 43–61%) (Figure 3B) were lower than the values based on intake calculation (Figure 3A), while the relative contributions of CP profiles in the air (12–37%) were higher than the values based on intake calculation. The fingerprinting approach cannot exclude CPs partitioning to food from air, which may contribute to an elevated estimation of contribution from the atmosphere. It is also possible that the intake calculation under- or overestimates the relative contributions of the exposure pathways.

Personalized samplers represent one of the future sampling strategies for complex environmental mixtures in humans.^48^ Personal air is representative for assessing personal exposure.^49^ In the current study, the personal air samples showed higher Pearson’s correlation coefficients (r) with the paired plasma samples than the stationary air samples (Table S8), and the use of results from personal air profiles instead of stationary air profiles (Figure 3C) seems to improve the performance of the forensic fingerprinting. The relative contributions of individual exposure pathways agree better with the results from the intake calculation (Figure 3A). The dietary intake was estimated to be 41–80% (median) of the total intake, and dermal exposure of LCCPs was 37% compared to 29% based on intake calculation (Table S9), but it is difficult to determine the statistical significance due to the limited number of personal air samples.

### Potential Sources

The concentrations of SCCPs, MCCPs, and LCCPs in the plasma samples were significantly correlated (*p* < 0.05, Table S11), which might be an indication that these mixtures often have been used together either intentionally, such as the CP technical mixtures produced with mixed chain length classes,^10,50^ or in the form of impurities.^12^ No significant differences in CP concentrations were observed between genders or in different age groups in the plasma (*p* < 0.05, Table S12).

Associations between CP concentrations in the diet samples and data from the food diaries were investigated (Table S12). Significantly higher SCCP concentrations were shown when the diet contained butter (*p* < 0.05) and significantly higher MCCP concentrations were shown when the diet contained eggs (*p* < 0.05). This seems consistent with a study on poultry in which laying hens showed high accumulation and transfer ratios of CPs.^51^ The median levels of CPs were slightly higher in the diet containing meat, but the difference was not statistically significant. Participants who consumed egg, butter, or meat more frequently showed slightly higher median plasma concentrations of CPs, but the differences were not statistically significant. This could possibly be due to the ubiquity of CPs in the human diet. Apart from the high DFs in food in the current cohort study, CPs have also been found in various food items sold on the European market^13,26,52^ and CPs have been found to migrate from food packaging into the food,^53^ or contaminate food during food processing (Figure 1).^13^ These possible dietary sources were not included in this study.

A few residence construction parameters showed significant correlations with the plasma CP levels (Table S12). Participants living in buildings built between 1952 and 2002 had higher levels of SCCPs than those living in older or newer buildings (Kruskal–Wallis tests, *p* < 0.05). This mirrors the time period of highest use of CPs in the Nordic countries and the ban of SCCPs in 2002 in Norway.^54^ This may also be in line with a study of older buildings in Germany where high contents of SCCPs were found in window sealing materials.^55^ CPs have also been found in insulation materials in the Netherlands.^29^ Participants living in homes with wooden floors had the lowest plasma CP levels, while the three participants having synthetic flooring had the highest plasma CP levels. This is reasonable, as CPs are widely used in items that contain or are made of plastic or rubber, such as in synthetic flooring materials.^56^ We explored associations with home items recorded in the questionnaire and found significantly higher plasma levels of SCCPs and MCCPs in participants owning sofas (Mann–Whitney *U* test, *p* < 0.05).

### Global Perspective on Human Exposure to CPs

Versatile uses of CPs consequently lead to diverse sources of human exposure to the chemicals. The C_*n*_Cl_*m*_ fingerprints provide forensic support in the identification of possible exposure sources. However, the application of the forensic approach requires both internal and multiple external exposure fingerprints. The application of the intake calculation approach does not require internal exposure data, and the calculation of CP intake based on the major CP contributor could promptly provide a preliminary exposure assessment. Among multiple exposure media, the diet contained very low absolute concentrations of CPs (median 23 ng/g ww which were ∼1‰ of the median dust concentration of 37 000 ng/g^27^) but contributed the most to the pollutant body burden as the amount of food consumed per day is high compared to ingestion of dust.

**Figure 4:**
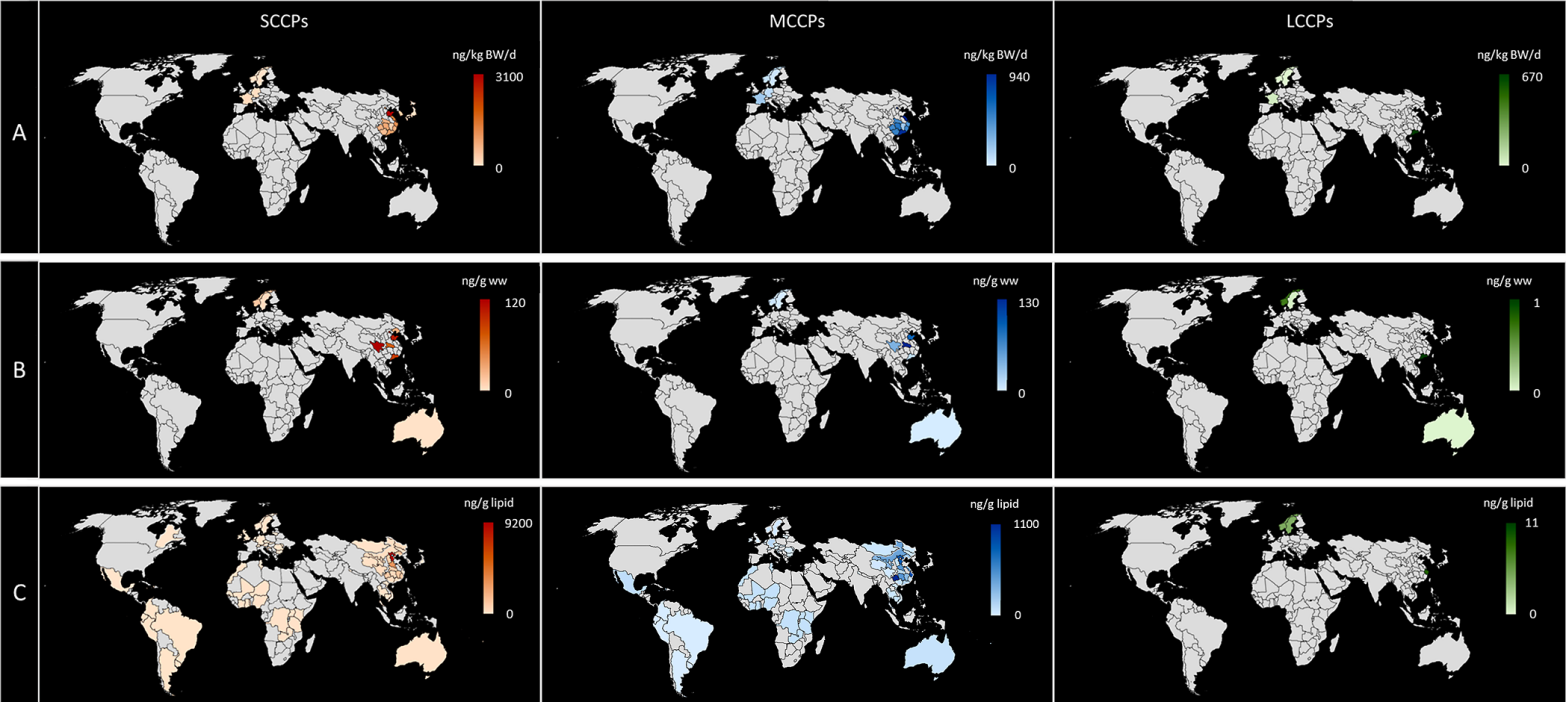
| Global levels of dietary intake (A), blood concentrations (B), and human milk concentrations (C) of SCCPs, MCCPs, and LCCPs. Data are plotted at a resolution of state/province if available. For tabular summaries, see Tables S5 and S6.

This cohort study from Norway adds to the growing database of human external and internal exposure to CPs, and shows this is unfortunately not a local case. To illustrate this, the dietary intakes, blood concentrations, and human milk concentrations of SCCPs, MCCPs, and LCCPs from the current cohort have been compared to those available from around the globe based on CP concentrations reported in the literature. The data are summarized in Tables S5 and S6 and visualized in Figure 4. The majority of the dietary intake and blood concentration data are available from China and several European countries, while major data gaps are present in the rest of the world. As the major producer of CPs, China reported generally higher levels of CPs in multiple matrices than the studied European countries. Mean SCCP concentration in the diet study of Jinan, China (3109 ng/kg BW/d) was above the RfD of 2300 ng/kg BW/d. Although the mean/median dietary intakes in most studies (Table S5) were below the RfDs, the 95th percentile exceeded the RfDs in certain cases, given the large variance among individuals. The variance can be reflected by the ratios between the 95th percentile and the mean/median intakes, which were between 2.5 and 4.5 in the present study (Table 1). In contrast to human plasma levels, mothers’ milk data are available from most of the regions worldwide, which demonstrates the advantage of noninvasive biomonitoring samples.

The global ban of SCCPs has further increased the use and emissions of MCCPs and LCCPs. For example, LCCPs have recently been found to be the predominant CP class in the German environment.^57^ Such trends may indicate a shift in collateral transport of SCCPs via food trade^58^ toward MCCPs and LCCPs, as well as a corresponding increase in human exposure to these CP classes via various exposure pathways such as dust ingestion and dermal exposure. However, data for LCCPs are severely lacking in the global database for all matrices (Figure 4). The current study raises a strong need for global attention to CPs, a large group of global contaminants, and their impacts on human beings and ecosystems.

## Supporting Information Available

References for Figure 1, sample list, reference standards, CP properties, global diet exposure to CPs, global human biomonitoring of CPs, statistical analyses, predicted versus measured plasma concentrations, top 10 and bottom 10 plasma levels; sample collection, sample pretreatment, intake estimation instrument settings, CP quantification, BL calculation, forensic fingerprinting, method validation, and CP fingerprints (PDF)
