# Supplementary material for: Dietary Intake Contributed the Most to Chlorinated Paraffin Body Burden in a Norwegian Cohort
Source: Environ Sci Technol. 2022 Nov 15;56(23):17080–9. doi: 10.1021/acs.est.2c04998 (PMC9730849; doi:10.1021/acs.est.2c04998)
Supplement: Supplementary file 1 — es2c04998_si_001.pdf [file es2c04998_si_001.pdf]

Supporting Information

Dietary Intake Contributed the Most to Chlorinated Paraffin Body Burden in a Norwegian Cohort

Bo Yuan,[1,*] Line Småstuen Haug,[2] Joo Hui Tay,[1,†] Juan Antonio Padilla-Sánchez,[2] Eleni Papadopoulou,[2] and Cynthia A. de Wit[1]

[1] Department of Environmental Science, Stockholm University, SE-10691 Stockholm, Sweden

[2] Department for Food Safety, Norwegian Institute of Public Health, NO-0213 Oslo, Norway

[†] Current address: Faculty of Industrial Sciences & Technology (FIST), Universiti Malaysia Pahang (UMP), Lebuhraya Tun Razak, 26300 Gambang, Kuantan, Pahang, Malaysia.

[*]Corresponding author: bo.yuan@aces.su.se; bo.yuan@ntnu.no

Pages: 26

Tables: 12

Texts: 7

Figures: 2

Table of Contents

Table S1. Environmental fate and exposure pathways of CPs and references 3

Table S2. Overview of samples available for the cohort study on exposure to CPs. 4

Table S3. CP reference mixture list 5

Table S4. Estimated physical-chemical properties and bioaccessibility data of CPs used for dietary exposure assessment 5

Table S5. Comparisons of human adult exposure to CPs via dietary intake between countries. 6

Table S6. Global human biomonitoring of CPs (ng/g lipid). 7

Table S7. Pearson correlation coefficient (*r*) between CP levels in matched human plasma and CP intakes from external exposure media 10

Table S8. Pearson correlation coefficient (*r*) between CP concentrations in plasma (ng/g lipid) and their parent CPs in stationary air (ng/m^3) and personal air (ng/m^3). 10

Table S9. Intakes calculated for different exposure pathways and predicted plasma concentrations derived from intake data compared to measured plasma concentrations. 11

Table S10. Comparisons of CP intakes and compositions between the participants with the 10 highest CP plasma levels and the rest of the cohort, as well as between those with the 10 lowest CP plasma levels and the rest of the cohort. 12

Table S11. Spearman rank correlations (*r*) between CP classes in plasma 12

Table S12. Statistical comparisons between characteristics of the study group/diet type/residential environment based on questionnaire answers and median concentrations of CPs (with detection frequencies above 75%) in the diet (ng/g ww) and plasma samples (ng/g lipid). 13

Text S1: Sample collection 15

Text S2: Sample Preparation 16

Text S3: MS settings and CP quantification 17

Text S4: Total exposure calculation from external media 18

Text S5: Calculation of Body Lipid (BL) 19

Text S6: Forensic Fingerprinting 19

Text S7: Determination of Chlorinated Paraffin in Human Serum 20

Figure S1. Forensic fingerprinting CP homologues in a plasma sample 21

Figure S2. Mean relative abundance of CP homologues with standard deviation error bars 22

References 23

Table S1. Environmental fate and exposure pathways of CPs and references

Fate and Pathway	Description	Reference
Dietary exposure	Dietary exposure via a wide range of food types	Table S4
Dietary exposure	Dietary exposure via vitamin E supplementary	Sprengel et al. (2019)[1]
Livestock uptake from feed	CPs were found in animal feed materials	Dong et al. (2019)[2]
Bioaccumulation of living organism	Accumulation from the environment and the feed	Castro et al. (2018)[3]
Plant uptake from soil and air	Accumulation in paddy rice via soil and air	Yuan et al. (2017)[4]
Plant uptake from soil	Accumulation in maize plants via agricultural soil	Chen et al. (2021)[5]
Contamination during cooking process	CPs were found high levels in the components of baking ovens	Gallistl et al. (2018)[6]
Contamination during cooking process	Leakage from hand blender use.	Yuan et al. (2017)[7]
Contamination from food packaging	Migration from plastic food packaging into food	Wang et al. (2019)[8]
Dermal exposure	Percutaneous penetration	Gao et al. (2021)[9]
Dust ingestion and inhalation exposure	CPs were found in indoor dust and air	Friden et al. (2011)[10]
Emissions due to industrial activities	Dispersion of CPs from a production plant to the surroundings	Xu et al. (2016)[11]
Emissions due to waste disposal	Human exposure due to e-waste recycling activities	Chen et al. (2018)[12]
Emissions due to waste disposal	Dispersion from a wastewater treatment plant to the surroundings	Zeng et al. (2011)[13]

Table S2. Overview of samples available for the cohort study on exposure to CPs.

Sample type	*Sample size*	*The samples were analyzed in*
**Human plasma**	59	present study
**Diet**	59	present study
**Hand wipe**	60	Yuan et al. (2020)[14]
**Settled dust**	61	Yuan et al. (2021)[15]
**Floor dust**	5	Yuan et al. (2021)[15]
**Stationary air**	61	Yuan et al. (2021)[15]
**Personal air**	13	Yuan et al. (2021)[15]

Table S3. CP reference mixture list.

Category	*Product*	*Manufacturer*	*Country*	*Ingredients**
**SCCPs**	C_{10} 50.18 %Cl	Ehrenstorfer GmbH	Germany	C_{10} 50.18% Cl
	C_{10} 55.00 %Cl	Ehrenstorfer GmbH	Germany	C_{10} 55.00% Cl
	C_{10} 60.09 %Cl	Ehrenstorfer GmbH	Germany	C_{10} 60.09% Cl
	C_{11} 50.21 %Cl	Ehrenstorfer GmbH	Germany	C_{11} 50.21% Cl
	Witaclor 149	Dynamit Nobel AG	Germany	C_{10-13} 49% Cl
	SCCP 51.5 %Cl	Ehrenstorfer GmbH	Germany	C_{10-13} 51.5% Cl
	SCCP 55.5 %Cl	Ehrenstorfer GmbH	Germany	C_{10-13} 55.5% Cl
	SCCP 63.0 %Cl	Ehrenstorfer GmbH	Germany	C_{10-13} 63.0% Cl
	Hüls 70C	Hüls AG	Germany	C_{10-13} 70% Cl
**MCCPs**	MCCP 42.0 %Cl	Ehrenstorfer GmbH	Germany	C_{14-17} 42.0% Cl
	MCCP 52.0 %Cl	Ehrenstorfer GmbH	Germany	C_{14-17} 52.0% Cl
	MCCP 57.0 %Cl	Ehrenstorfer GmbH	Germany	C_{14-17} 57.0% Cl
	Cloparin 49st	Caffaro	Italy	C_{14-17} 49% Cl
	Cloparin 50	Caffaro	Italy	C_{14-17} 50% Cl
	Cereclor S52	INEOS Chlor Ltd.	UK	C_{14-17} 52% Cl
**LCCPs**	Hüls 40N	Hüls AG	Germany	C_{18-26} 40% Cl
	Witaclor 549	Dynamit Nobel AG	Germany	C_{18-25} 49% Cl
	Uniclor40	Neville Chemical Co	USA	C_{22-27} 40% Cl
	LCCP 36.0 %Cl	Ehrenstorfer GmbH	Germany	C_{18-20} 36.0% Cl
	LCCP 49.0 %Cl	Ehrenstorfer GmbH	Germany	C_{18-20} 49.0% Cl
**Unknown**	CP-52	Unknown	China	C_{6-29} 52% Cl[†]

* specifications from the manufacturer.

† determined using an APCI-QTOF; not specified by the manufacturer.

Table S4. Estimated physical-chemical properties and bioaccessibility data of CPs used for dietary exposure assessment

	**Example molecular weight**	**Log K_{OW}**	**Log K_{OA}**	**Estimated AF_{dermal}[14]**	**Bioavailability data**	**Estimated $AF_{ingestion/dietary}$**	**Estimated half-life (year)**
**vSCCPs**	335 ($C_9H_{14}Cl_6$)	5.99[16]	7.24[16]	0.27	–	0.33[*]	–
**SCCPs**	363 ($C_{11}H_{18}Cl_6$)	4.10-8.67[17]	9-11[18]	0.27	0.211[19]	0.211	5.1[20]
**MCCPs**	405 ($C_{14}H_{24}Cl_6$)<br>516 ($C_{17}H_{28}Cl_8$)	5.56-8.38[17]	11-15[18]	0.34	0.079[19]	0.079	1.2[20]
**LCCPs**	461 ($C_{18}H_{32}Cl_6$)<br>545 ($C_{24}H_{44}Cl_6$)<br>713 ($C_{36}H_{68}Cl_6$)	6.58-11.34[17]	–	0.13	0.03[**]	0.03	0.6[20]

*Adopted from BDE-28 which has similar molecular weight (406.9) and Log K_{OW} (5.94)[21].
** predicted on the basis of the regression model[19] that the bioaccessibility of homologue $C_{18}Cl_7$ was ~33% of $C_{14}Cl_7$ (the most abundant LCCP and MCCP homologues, respectively, in the blood and food samples of the present study, see Figure S1).

Table S5. Comparisons of human adult exposure to CPs (ng/kg BW/d) via dietary intake between countries.

Region	Year	Food Type	Population	CP class	Dietary intake (ng/kg BW/d)			Reference
					mean/GM	median	95th Percentile	
Oslo, Norway	2013	duplicate diet	Cohort of 61 adult participants	SCCPs	43	42	120	present study
Oslo, Norway	2013	duplicate diet	Cohort of 61 adult participants	MCCPs	96	96	250	present study
Oslo, Norway	2013	duplicate diet	Cohort of 61 adult participants	LCCPs	8.9	8.5	38	present study
German online market		dietary supplements	European adult	SCCPs			27	Sprengel et al. (2019)[1]
German online market		dietary supplements	European adult	MCCPs			140	Sprengel et al. (2019)[1]
South Germany, Baden-Württemberg	2018.09-2019.08	market basket	Adults (18-64 years)	SCCPs	100			Krätschmer et al. (2021)[22]
South Germany, Baden-Württemberg	2018.09-2019.08	market basket	Adults (18-64 years)	MCCPs	100			Krätschmer et al. (2021)[22]
South Germany, Baden-Württemberg	2018.09-2019.08	ready-made meals	Adults (18-64 years)	SCCPs	57			Krätschmer et al. (2021)[22]
South Germany, Baden-Württemberg	2018.09-2019.08	ready-made meals	Adults (18-64 years)	MCCPs	35			Krätschmer et al. (2021)[22]
South Germany, Baden-Württemberg	2018.09-2019.08	total diet samples_balanced diet	Adults (18-64 years)	SCCPs	63			Krätschmer et al. (2021)[22]
South Germany, Baden-Württemberg	2018.09-2019.08	total diet samples_balanced diet	Adults (18-64 years)	MCCPs	54			Krätschmer et al. (2021)[22]
South Germany, Baden-Württemberg	2018.09-2019.08	total diet samples_vegetarian diet	Adults (18-64 years)	SCCPs	72			Krätschmer et al. (2021)[22]
South Germany, Baden-Württemberg	2018.09-2019.08	total diet samples_vegetarian diet	Adults (18-64 years)	MCCPs	120			Krätschmer et al. (2021)[22]
Germany	2014-2017	salmon sold on the market	20- years	S+MCCPs	0.87	0.68	2.5	Krätschmer et al. (2019)[23]
Nantes, France	2019-10	6 food categories, market basket	18-79 years old, 73.6 kg	SCCPs	135			Mézière (2020)[24]
Nantes, France	2019-10	6 food categories, market basket	18-79 years old, 73.6 kg	MCCPs	175			Mézière (2020)[24]
Nantes, France	2019-10	6 food categories, market basket	18-79 years old, 73.6 kg	LCCPs*	37			Mézière (2020)[24]
Sweden	2015	market basket	local adult, 76.6 kg	SCCPs	18			Yuan et al. (2017)[7]
Sweden	2015	market basket	local adult, 76.6 kg	MCCPs	39			Yuan et al. (2017)[7]
Sweden	2015	market basket	local adult, 76.6 kg	LCCPs	2			Yuan et al. (2017)[7]
Japan	2005	market basket	30-39 years, female	SCCPs		110	210	Iino et al. (2005)[25]
Japan	2005	market basket	30-39 years, male	SCCPs		110	220	Iino et al. (2005)[25]
Japan	2009	duplicate diet, n=40	26-29 years	SCCPs		620	1200	Harada et al. (2011)[26]
Korea	2016-2019	59 food species	female (19-49y, BW=58 kg)	SCCPs		781		Lee et al. (2020)[27]
Korea	2016-2019	59 food species	male (19-49y, BW=74.5 kg)	SCCPs		888		Lee et al. (2020)[27]
Jinan, China	2019.04	40 food species	residents	SCCPs	3109		4357	Li et al. (2020)[28]
Beijing, China	2016	duplicate diet	21-31 yr male	SCCPs	611			Gao et al. (2018)[29]
Beijing, China	2016	duplicate diet	21-31 yr male	MCCPs	730			Gao et al. (2018)[29]
Beijing, China	2009	duplicate diet, n=10	local participants	SCCPs		54		Harada et al. (2011)[26]
20 provinces, China	2009	12 meat categories	18-45 years	SCCPs		130	560	Huang et al. (2018)[30]
20 provinces, China	2009	12 meat categories	18-45 years	MCCPs		4.7	31	Huang et al. (2018)[30]
Qingyuan, China	2013, 2016	home-produced eggs	adult	SCCPs	274			Zeng et al. (2018)[31]
Qingyuan, China	2013, 2016	home-produced eggs	adult	MCCPs	193			Zeng et al. (2018)[31]
Shanghai, China	2017-2018	7 food categories, total diet study	general population, 18-45 years old, men, 63 kg	SCCPs	420			Cui et al. (2020)[32]
Fujian, China	2017-2018	7 food categories, total diet study	general population, 18-45 years old, men, 63 kg	SCCPs	740			Cui et al. (2020)[32]
Jiangxi, China	2017-2018	7 food categories, total diet study	general population, 18-45 years old, men, 63 kg	SCCPs	260			Cui et al. (2020)[32]
Jiangsu, China	2017-2018	7 food categories, total diet study	general population, 18-45 years old, men, 63 kg	SCCPs	1300			Cui et al. (2020)[32]
Zhejiang, China	2017-2018	7 food categories, total diet study	general population, 18-45 years old, men, 63 kg	SCCPs	1100			Cui et al. (2020)[32]
Hubei, China	2017-2018	7 food categories, total diet study	general population, 18-45 years old, men, 63 kg	SCCPs	640			Cui et al. (2020)[32]
Guangxi, China	2017-2018	7 food categories, total diet study	general population, 18-45 years old, men, 63 kg	SCCPs	530			Cui et al. (2020)[32]
Hunan, China	2017-2018	7 food categories, total diet study	general population, 18-45 years old, men, 63 kg	SCCPs	800			Cui et al. (2020)[32]
Guizhou, China	2017-2018	7 food categories, total diet study	general population, 18-45 years old, men, 63 kg	SCCPs	660			Cui et al. (2020)[32]
Shanghai, China	2017-2018	7 food categories, total diet study	general population, 18-45 years old, men, 63 kg	MCCPs	190			Cui et al. (2020)[32]
Fujian, China	2017-2018	7 food categories, total diet study	general population, 18-45 years old, men, 63 kg	MCCPs	500			Cui et al. (2020)[32]
Jiangxi, China	2017-2018	7 food categories, total diet study	general population, 18-45 years old, men, 63 kg	MCCPs	180			Cui et al. (2020)[32]
Jiangsu, China	2017-2018	7 food categories, total diet study	general population, 18-45 years old, men, 63 kg	MCCPs	940			Cui et al. (2020)[32]
Zhejiang, China	2017-2018	7 food categories, total diet study	general population, 18-45 years old, men, 63 kg	MCCPs	430			Cui et al. (2020)[32]
Hubei, China	2017-2018	7 food categories, total diet study	general population, 18-45 years old, men, 63 kg	MCCPs	460			Cui et al. (2020)[32]
Guangxi, China	2017-2018	7 food categories, total diet study	general population, 18-45 years old, men, 63 kg	MCCPs	520			Cui et al. (2020)[32]
Hunan, China	2017-2018	7 food categories, total diet study	general population, 18-45 years old, men, 63 kg	MCCPs	570			Cui et al. (2020)[32]
Guizhou, China	2017-2018	7 food categories, total diet study	general population, 18-45 years old, men, 63 kg	MCCPs	480			Cui et al. (2020)[32]
Shenzhen, China		6 food categories	59 kg	SCCPs	897			Dong et al. (2020)[20]
Shenzhen, China		6 food categories	59 kg	MCCPs	910			Dong et al. (2020)[20]
Shenzhen, China		6 food categories	59 kg	LCCPs	674			Dong et al. (2020)[20]

*without vLCCPs

Table S6. Global human biomonitoring of CPs (ng/g lipid).

Region	Year	Biomonitoring Type	Sample info	CP class	Concentration (ng/g lipid)				Reference
					mean	min	median	max	
Norway	2013	serum	59 adults	SCCPs		<520	2460	10407	present study
Norway	2013	serum	59 adults	MCCPs		<590	1097	9772	present study
Norway	2013	serum	59 adults	LCCPs		<51	121	696	present study
Sweden	2019	serum	2 females	SCCPs			790		Bergman et al. (2022)[33]
Sweden	2019	serum	2 females	MCCPs			520		Bergman et al. (2022)[33]
Sweden	2019	serum	2 females	LCCPs			16		Bergman et al. (2022)[33]
Southeast Queensland, Australia	2004-2015	serum	0-60 years, pool	SCCPs	102	<MDL	<MDL	140	van Mourik et al. (2020)[34]
Southeast Queensland, Australia	2004-2015	serum	0-60 years, pool	MCCPs	230	<MDL	190	520	van Mourik et al. (2020)[34]
Southeast Queensland, Australia	2004-2015	serum	0-60 years, pool	LCCPs	<MDL	<MDL	<MDL	<MDL	van Mourik et al. (2020)[34]
Beijing, China	2013.02-2013.05	serum	maternal, individual	SCCPs	18100	2570	16100	57800	Qiao et al. (2018)[35]
Beijing, China	2013.02-2013.05	serum	maternal, individual	MCCPs	1760	478	1340	6400	Qiao et al. (2018)[35]
Shenzhen, China	2012.08	venous blood	24-45 years	SCCPs		370	3500	35000	Li et al. (2017)[36]
Shenzhen, China	2012.08	venous blood	24-45 years	MCCPs		130	740	3200	Li et al. (2017)[36]
Shenzhen, China	2012.08	venous blood	24-45 years	LCCPs		22	150	530	Li et al. (2017)[36]
Guangzhou, China	2016.12-2017.03	plasma	maternal, individual	SCCPs	889	407	880	1570	Chen et al. (2020)[37]
Guangzhou, China	2016.12-2017.03	plasma	maternal, individual	MCCPs	464	199	421	851	Chen et al. (2020)[37]
Wuhan, China[a]	2015.11-2016.03	serum	maternal, individual	SCCPs		15.9	66.2	584	Aamir et al. (2019)[38]
Wuhan, China[a]	2015.11-2016.03	serum	maternal, individual	MCCPs		29.3	126	1006	Aamir et al. (2019)[38]
Mianyang, China[a]	2018.04	serum	maternal, individual	SCCPs			117.1		Liu et al. (2020)[39]
Mianyang, China[a]	2018.04	serum	maternal, individual	MCCPs			38.9		Liu et al. (2020)[39]
Jinan, China	2019.09-12	serum	>18 years old	SCCPs	23000		12200		Zhao et al. (2021)[40]
Jinan, China	2019.09-12	serum	>18 years old	MCCPs	18200		11200		Zhao et al. (2021)[40]
Dalian, China[a]	2015-10	plasma	50 participants, 10-88yr	SCCPs	32	<LOD	26.40	203.00	Xu et al. (2019)[41]
Stockholm	2011, 2016	individual milk	mean 29 years old	SCCPs		<LOD	14	27.8	Zhou et al. (2020)[42]
Stockholm	2011, 2016	individual milk	mean 29 years old	MCCPs		<LOD	27.4	77.7	Zhou et al. (2020)[42]
Stockholm	2011, 2016	individual milk	mean 29 years old	LCCPs		<LOD	4.14	21.4	Zhou et al. (2020)[42]
Bodø, Norway	2014	individual milk	mean 35 years old	SCCPs		<LOD	10.2	120	Zhou et al. (2020)[42]
Bodø, Norway	2014	individual milk	mean 35 years old	MCCPs		<LOD	43.1	311	Zhou et al. (2020)[42]
Bodø, Norway	2014	individual milk	mean 35 years old	LCCPs		<LOD	4.26	29	Zhou et al. (2020)[42]
Quebec, Canada				SCCPs		11		17	Tomy (1997)[43]
Lancaster, UK	2001-2002	individual milk		SCCPs		49	180	820	Thomas et al. (2006)[44]
Lancaster, UK	2001-2002	individual milk		MCCPs		6.2	21	320	Thomas et al. (2006)[44]
Seoul, South Korea [b]	2007-2010	pooled milk	30.9 ± 3.4 yr	SCCPs	<20			3.4	Cao et al. (2017)[45]
Busan, South Korea [c]	2008-2009	pooled milk	31.7 ± 4.2 yr	SCCPs	<20				Cao et al. (2017)[45]
Kyoto, Japan [c]	2009-2010	pooled milk	32.3 ± 4.4 yr	SCCPs	<20				Cao et al. (2017)[45]
Sendai, Japan [b]	2007-2009	pooled milk	32.4 ± 4.0 yr	SCCPs	<20			17.5	Cao et al. (2017)[45]
Africa[d]	2012-2019	pooled milk	<30 y, primiparity	SCCPs		40	68	310	Krätschmer et al. (2021)[46]
Asia[d] (except Mainland China)	2012-2019	pooled milk	<30 y, primiparity	SCCPs		27	59	160	Krätschmer et al. (2021)[46]
Europe[d]	2012-2019	pooled milk	<30 y, primiparity	SCCPs		9.8	33	100	Krätschmer et al. (2021)[46]
Centrial America and Caribbean[d]	2012-2019	pooled milk	<30 y, primiparity	SCCPs		28	38	48	Krätschmer et al. (2021)[46]
Oceania[d]	2012-2019	pooled milk	<30 y, primiparity	SCCPs		36	86	86	Krätschmer et al. (2021)[46]
South America[d]	2012-2019	pooled milk	<30 y, primiparity	SCCPs		17	41	41	Krätschmer et al. (2021)[46]
Africa[d]	2012-2019	pooled milk	<30 y, primiparity	MCCPs		47	94	370	Krätschmer et al. (2021)[46]
Asia[d] (except Mainland China)	2012-2019	pooled milk	<30 y, primiparity	MCCPs		38	69	540	Krätschmer et al. (2021)[46]
Europe[d]	2012-2019	pooled milk	<30 y, primiparity	MCCPs		19	31	110	Krätschmer et al. (2021)[46]
Centrial America and Caribbean[d]	2012-2019	pooled milk	<30 y, primiparity	MCCPs		46	58	110	Krätschmer et al. (2021)[46]
Oceania[d]	2012-2019	pooled milk	<30 y, primiparity	MCCPs		34	87	200	Krätschmer et al. (2021)[46]
South America[d]	2012-2019	pooled milk	<30 y, primiparity	MCCPs		5.6	28	140	Krätschmer et al. (2021)[46]
China									
Heilongjiang	2011	pooled milk	urban, mean 26.5yr	SCCPs	489				Xia et al. (2017)[47]
Jilin	2011	pooled milk	urban, mean 26.5yr	SCCPs	368				Xia et al. (2017)[47]
Liaoning	2011	pooled milk	urban, mean 26.5yr	SCCPs	2180				Xia et al. (2017)[47]
Neimenggu	2011	pooled milk	urban, mean 26.5yr	SCCPs	131				Xia et al. (2017)[47]
Hebei	2011	pooled milk	urban, mean 26.5yr	SCCPs	16100				Xia et al. (2017)[47]
Ningxia	2011	pooled milk	urban, mean 26.5yr	SCCPs	478				Xia et al. (2017)[47]
Qinghai	2011	pooled milk	urban, mean 26.5yr	SCCPs	203				Xia et al. (2017)[47]

Henan	2011	pooled milk	urban, mean 26.5yr	SCCPs	7060				Xia et al. (2017)[47]
Shanxi	2011	pooled milk	urban, mean 26.5yr	SCCPs	977				Xia et al. (2017)[47]
Shanghai	2011	pooled milk	urban, mean 26.5yr	SCCPs	2530				Xia et al. (2017)[47]
Sichuan	2011	pooled milk	urban, mean 26.5yr	SCCPs	465				Xia et al. (2017)[47]
Hubei	2011	pooled milk	urban, mean 26.5yr	SCCPs	1180				Xia et al. (2017)[47]
Jiangxi	2011	pooled milk	urban, mean 26.5yr	SCCPs	1981				Xia et al. (2017)[47]
Fujian	2011	pooled milk	urban, mean 26.5yr	SCCPs	1650				Xia et al. (2017)[47]
Guangxi	2011	pooled milk	urban, mean 26.5yr	SCCPs	342				Xia et al. (2017)[47]
Guangdong	2011	pooled milk	urban, mean 26.5yr	SCCPs	355				Xia et al. (2017)[47]
Heilongjiang	2011	pooled milk	urban, mean 26.5yr	MCCPs	28.5				Xia et al. (2017)[47]
Jilin	2011	pooled milk	urban, mean 26.5yr	MCCPs	63.9				Xia et al. (2017)[47]
Liaoning	2011	pooled milk	urban, mean 26.5yr	MCCPs	409				Xia et al. (2017)[47]
Neimenggu	2011	pooled milk	urban, mean 26.5yr	MCCPs	22.3				Xia et al. (2017)[47]
Hebei	2011	pooled milk	urban, mean 26.5yr	MCCPs	1501				Xia et al. (2017)[47]
Ningxia	2011	pooled milk	urban, mean 26.5yr	MCCPs	40.2				Xia et al. (2017)[47]
Qinghai	2011	pooled milk	urban, mean 26.5yr	MCCPs	47				Xia et al. (2017)[47]
Henan	2011	pooled milk	urban, mean 26.5yr	MCCPs	736				Xia et al. (2017)[47]
Shanxi	2011	pooled milk	urban, mean 26.5yr	MCCPs	64.8				Xia et al. (2017)[47]
Shanghai	2011	pooled milk	urban, mean 26.5yr	MCCPs	355.7				Xia et al. (2017)[47]
Sichuan	2011	pooled milk	urban, mean 26.5yr	MCCPs	37.3				Xia et al. (2017)[47]
Hubei	2011	pooled milk	urban, mean 26.5yr	MCCPs	108				Xia et al. (2017)[47]
Jiangxi	2011	pooled milk	urban, mean 26.5yr	MCCPs	126				Xia et al. (2017)[47]
Fujian	2011	pooled milk	urban, mean 26.5yr	MCCPs	102				Xia et al. (2017)[47]
Guangxi	2011	pooled milk	urban, mean 26.5yr	MCCPs	43.5				Xia et al. (2017)[47]
Guangdong	2011	pooled milk	urban, mean 26.5yr	MCCPs	47.5				Xia et al. (2017)[47]
Heilongjiang	2011	pooled milk	rural, 17-39y, mean 26	SCCPs	217				Xia et al. (2017)[48]
Hebei	2011	pooled milk	rural, 17-39y, mean 26	SCCPs	2310				Xia et al. (2017)[48]
Ningxia	2011	pooled milk	rural, 17-39y, mean 26	SCCPs	147				Xia et al. (2017)[48]
Henan	2011	pooled milk	rural, 17-39y, mean 26	SCCPs	975				Xia et al. (2017)[48]
Shanxi	2011	pooled milk	rural, 17-39y, mean 26	SCCPs	784				Xia et al. (2017)[48]
Shanghai	2011	pooled milk	rural, 17-39y, mean 26	SCCPs	2130				Xia et al. (2017)[48]
Hubei	2011	pooled milk	rural, 17-39y, mean 26	SCCPs	524				Xia et al. (2017)[48]
Fujian	2011	pooled milk	rural, 17-39y, mean 26	SCCPs	503				Xia et al. (2017)[48]
Jilin	2011	pooled milk	rural, 17-39y, mean 26	SCCPs	143				Xia et al. (2017)[48]
Liaoning	2011	pooled milk	rural, 17-39y, mean 26	SCCPs	649				Xia et al. (2017)[48]
Neimenggu	2011	pooled milk	rural, 17-39y, mean 26	SCCPs	75.4				Xia et al. (2017)[48]
Qinghai	2011	pooled milk	rural, 17-39y, mean 26	SCCPs	65.6				Xia et al. (2017)[48]
Sichuan	2011	pooled milk	rural, 17-39y, mean 26	SCCPs	175				Xia et al. (2017)[48]
Jiangxi	2011	pooled milk	rural, 17-39y, mean 26	SCCPs	840				Xia et al. (2017)[48]
Guangxi	2011	pooled milk	rural, 17-39y, mean 26	SCCPs	89.8				Xia et al. (2017)[48]
Guangdong	2011	pooled milk	rural, 17-39y, mean 26	SCCPs	77.6				Xia et al. (2017)[48]
Heilongjiang	2011	pooled milk	rural, 17-39y, mean 26	MCCPs	15.3				Xia et al. (2017)[48]
Hebei	2011	pooled milk	rural, 17-39y, mean 26	MCCPs	146				Xia et al. (2017)[48]
Ningxia	2011	pooled milk	rural, 17-39y, mean 26	MCCPs	29				Xia et al. (2017)[48]
Henan	2011	pooled milk	rural, 17-39y, mean 26	MCCPs	80.8				Xia et al. (2017)[48]
Shanxi	2011	pooled milk	rural, 17-39y, mean 26	MCCPs	68.1				Xia et al. (2017)[48]
Shanghai	2011	pooled milk	rural, 17-39y, mean 26	MCCPs	100				Xia et al. (2017)[48]
Hubei	2011	pooled milk	rural, 17-39y, mean 26	MCCPs	113				Xia et al. (2017)[48]
Fujian	2011	pooled milk	rural, 17-39y, mean 26	MCCPs	55.4				Xia et al. (2017)[48]
Jilin	2011	pooled milk	rural, 17-39y, mean 26	MCCPs	35.4				Xia et al. (2017)[48]
Liaoning	2011	pooled milk	rural, 17-39y, mean 26	MCCPs	65.1				Xia et al. (2017)[48]
Neimenggu	2011	pooled milk	rural, 17-39y, mean 26	MCCPs	15.1				Xia et al. (2017)[48]
Qinghai	2011	pooled milk	rural, 17-39y, mean 26	MCCPs	18.1				Xia et al. (2017)[48]
Sichuan	2011	pooled milk	rural, 17-39y, mean 26	MCCPs	12				Xia et al. (2017)[48]
Jiangxi	2011	pooled milk	rural, 17-39y, mean 26	MCCPs	58				Xia et al. (2017)[48]
Guangxi	2011	pooled milk	rural, 17-39y, mean 26	MCCPs	9.51				Xia et al. (2017)[48]
Guangdong	2011	pooled milk	rural, 17-39y, mean 26	MCCPs	21.6				Xia et al. (2017)[48]
Gansu	2017	pooled milk	urban	SCCPs	131				Xu et al. (2021)[49]
Inner Mongolia	2017	pooled milk	urban	SCCPs	417				Xu et al. (2021)[49]
Henan	2017	pooled milk	urban	SCCPs	808				Xu et al. (2021)[49]

Jiangsu	2017	pooled milk	urban	SCCPs	211				Xu et al. (2021)[49]
Shanghai	2017	pooled milk	urban	SCCPs	626				Xu et al. (2021)[49]
Jiangxi	2017	pooled milk	urban	SCCPs	633				Xu et al. (2021)[49]
Guangxi	2017	pooled milk	urban	SCCPs	138				Xu et al. (2021)[49]
Guangdong	2017	pooled milk	urban	SCCPs	406				Xu et al. (2021)[49]
Zhejiang	2017	pooled milk	urban	SCCPs	321				Xu et al. (2021)[49]
Hunan	2017	pooled milk	urban	SCCPs	380				Xu et al. (2021)[49]
Guizhou	2017	pooled milk	urban	SCCPs	–				Xu et al. (2021)[49]
Gansu	2017	pooled milk	urban	MCCPs	149				Xu et al. (2021)[49]
Inner Mongolia	2017	pooled milk	urban	MCCPs	1714				Xu et al. (2021)[49]
Henan	2017	pooled milk	urban	MCCPs	251				Xu et al. (2021)[49]
Jiangsu	2017	pooled milk	urban	MCCPs	430				Xu et al. (2021)[49]
Shanghai	2017	pooled milk	urban	MCCPs	569				Xu et al. (2021)[49]
Jiangxi	2017	pooled milk	urban	MCCPs	750				Xu et al. (2021)[49]
Guangxi	2017	pooled milk	urban	MCCPs	94				Xu et al. (2021)[49]
Guangdong	2017	pooled milk	urban	MCCPs	467				Xu et al. (2021)[49]
Zhejiang	2017	pooled milk	urban	MCCPs	905				Xu et al. (2021)[49]
Hunan	2017	pooled milk	urban	MCCPs	476				Xu et al. (2021)[49]
Guizhou	2017	pooled milk	urban	MCCPs	–				Xu et al. (2021)[49]
Gansu	2017	pooled milk	rural	SCCPs	513				Xu et al. (2021)[49]
Inner Mongolia	2017	pooled milk	urban	SCCPs	536				Xu et al. (2021)[49]
Henan	2017	pooled milk	urban	SCCPs	1543				Xu et al. (2021)[49]
Jiangsu	2017	pooled milk	urban	SCCPs	–				Xu et al. (2021)[49]
Shanghai	2017	pooled milk	urban	SCCPs	353				Xu et al. (2021)[49]
Jiangxi	2017	pooled milk	urban	SCCPs	139				Xu et al. (2021)[49]
Guangxi	2017	pooled milk	urban	SCCPs	635				Xu et al. (2021)[49]
Guangdong	2017	pooled milk	urban	SCCPs	189				Xu et al. (2021)[49]
Zhejiang	2017	pooled milk	urban	SCCPs	–				Xu et al. (2021)[49]
Hunan	2017	pooled milk	urban	SCCPs	–				Xu et al. (2021)[49]
Guizhou	2017	pooled milk	urban	SCCPs	988				Xu et al. (2021)[49]
Gansu	2017	pooled milk	urban	MCCPs	421				Xu et al. (2021)[49]
Inner Mongolia	2017	pooled milk	urban	MCCPs	642				Xu et al. (2021)[49]
Henan	2017	pooled milk	urban	MCCPs	963				Xu et al. (2021)[49]
Jiangsu	2017	pooled milk	urban	MCCPs	–				Xu et al. (2021)[49]
Shanghai	2017	pooled milk	urban	MCCPs	572				Xu et al. (2021)[49]
Jiangxi	2017	pooled milk	urban	MCCPs	561				Xu et al. (2021)[49]
Guangxi	2017	pooled milk	urban	MCCPs	237				Xu et al. (2021)[49]
Guangdong	2017	pooled milk	urban	MCCPs	211				Xu et al. (2021)[49]
Zhejiang	2017	pooled milk	urban	MCCPs	–				Xu et al. (2021)[49]
Hunan	2017	pooled milk	urban	MCCPs	–				Xu et al. (2021)[49]
Guizhou	2017	pooled milk	urban	MCCPs	1089				Xu et al. (2021)[49]
Beijing*	2007-2009	individual milk	26.8 ± 2.1 yr	SCCPs			28	66	Cao et al. (2017)[45]
Shanghai	2015-2016	individual milk	mean 29 yr	SCCPs		<LOD	35	676	Zhou et al. (2020)[42]
Jiaxing	2015-2016	individual milk	mean 33 yr	SCCPs		<LOD	28.6	642	Zhou et al. (2020)[42]
Shaoxing	2010	individual milk	20-25 yr	SCCPs		<LOD	37.9	124	Zhou et al. (2020)[42]
Zhejiang	2010, 2015-2016	individual milk	mean 20-33 yr	SCCPs		<LOD	34.9	642	Zhou et al. (2020)[42]
Shanghai	2015-2016	individual milk	mean 29 yr	MCCPs		38	70.1	1260	Zhou et al. (2020)[42]
Jiaxing	2015-2016	individual milk	mean 33 yr	MCCPs		<LOD	63	547	Zhou et al. (2020)[42]
Shaoxing	2010	individual milk	20-25 yr	MCCPs		38	121	187	Zhou et al. (2020)[42]
Zhejiang	2010, 2015-2016	individual milk	mean 20-33 yr	MCCPs		<LOD	87.9	547	Zhou et al. (2020)[42]
Shanghai	2015-2016	individual milk	mean 29 yr	LCCPs		<LOD	10.5	56.9	Zhou et al. (2020)[42]
Jiaxing	2015-2016	individual milk	mean 33 yr	LCCPs		<LOD	5.23	18.4	Zhou et al. (2020)[42]
Shaoxing	2010	individual milk	20-25 yr	LCCPs		4.9	14.6	184	Zhou et al. (2020)[42]
Zhejiang	2010, 2015-2016	individual milk	mean 20-33 yr	LCCPs		<LOD	8.36	184	Zhou et al. (2020)[42]

[a] unit: ng/g mL

[b] Summation of detected SCCP congeners at concentrations more than the lowest MDL is presented.

[c] When the sum of congeners was less than the highest MDL, the value was considered as not detected.

[d] The results of individual countries were not given in the publication[46]; therefore, all the sampled countries shared the same median concentration of the continent region in Figure 4, unless the countries[50] have their own reported concentrations.

Table S7. Pearson correlation coefficient (*r*) between CP levels in matched human plasma and CP intakes from external exposure media. The dataset was log-transformed before statistical testing.

	Plasma		
	SCCPs	MCCPs	LCCPs
Settled dust			
SCCPs	-0.05		
MCCPs		-0.11	
LCCPs			-0.05
Hand wipe			
SCCPs	-0.03		
MCCPs		0.05	
LCCPs			-0.09
Stationary air			
SCCPs	0.03		
MCCPs		-0.08	
LCCPs			-0.12
Diet			
SCCPs	0.38*		
MCCPs		0.28*	
LCCPs			0.36*

* $p < 0.05$

Table S8. Pearson correlation coefficient (*r*) between CP concentrations in plasma (ng/g lipid) and their parent CPs in stationary air (ng/m^3) and personal air (ng/m^3). The dataset was log-transformed before statistical testing.

	log (CPs) in plasma vs		
	SCCPs (n = 12)	MCCPs (n = 11)	LCCPs (n = 11)
log (CPs) in personal air			
SCCPs	0.46		
MCCPs		0.55*	
LCCPs			0.13
log (CPs) in paired stationary air			
SCCPs	0.37		
MCCPs		0.01	
LCCPs			0.55

* $p = 0.05$

Table S9. Intakes calculated for different exposure pathways and predicted plasma concentrations derived from intake data compared to measured plasma concentrations.

CP category	vSCCPs	SCCPs	MCCPs	LCCPs
**Median intake (ng/kg BW/d)**				
Dust ingestion[15]	0.00069	0.58	0.70	0.090
Inhalation[15]	0.073	1.6	0.23	0.0046
Dermal uptake[14]	–*	0.62	2.4	0.3
Dietary intake	1.9	18	15	0.51
**Predicted plasma concentration (ng/g lipid)**				
median	–*	2100	410	13
mean	–*	2600	510	28
**Measured plasma concentration (ng/g lipid)**				
median	68	2500	1100	120
mean	92	3000	2100	180
**Predicted concentration ÷ (paired) measured concentration**				
median	–*	1.0	0.48	0.15
mean	–*	1.5	0.70	0.67
**Predicted concentration ÷ (paired) measured concentration, using personal air**				
median	–*	1.0	0.5	0.20
mean	–*	1.2	1.2	0.26
**Relative contribution of external pathway based on the intake results (median**)**				
Dust ingestion	–*	2.2%	3.3%	10%
Inhalation	–*	6.5%	0.90%	0.38%
Dermal uptake	–*	2.9%	14%	29%
Dietary intake	–*	88%	82%	60%
**Relative contribution of external pathway based on the intake results (mean)**				
Dust ingestion	–*	5.4%	5.9%	12%
Inhalation	–*	11%	2.1%	1.5%
Dermal uptake	–*	4.8%	18%	34%
Dietary intake	–*	79%	74%	52%
**Relative contribution of external pathway based on forensic fingerprinting (median**)**				
Dust ingestion	–*	0.0%	15%	38%
Inhalation	–*	37%	30%	12%
Dermal uptake	–*	2.0%	0.30%	7.2%
Dietary intake	–*	61%	55%	43%
**Relative contribution of external pathway based on forensic fingerprinting (mean)**				
Dust ingestion	–*	2.5%	18%	33%
Inhalation	–*	41%	26%	24%
Dermal uptake	–*	15%	18%	7.1%
Dietary intake	–*	42%	39%	36%
**Relative contribution of external pathway based on forensic fingerprinting and personal air (median**)**				
Dust ingestion	–*	0.0%	0.0%	18%
Inhalation	–*	20%	38%	3.6%
Dermal uptake	–*	0.0%	7.5%	37%
Dietary intake	–*	80%	54%	41%
**Relative contribution of external pathway based on forensic fingerprinting and personal air (mean)**				
Dust ingestion	–*	1.3%	24%	19%
Inhalation	–*	30%	30%	14%
Dermal uptake	–*	9.0%	13%	31%
Dietary intake	–*	60%	32%	36%

*vSCCPs were not calculated/available due to a low detection frequency

** the medians of the relative contributions of individual samples have been normalized; before normalization, the sum of median relative contributions was not equal to 100% due to that the median relative contributions of all the four exposure pathways were not from the same participant.

Table S10. Comparisons of CP intakes and compositions between the participants with the 10 highest CP plasma levels and the rest of the cohort, as well as between those with the 10 lowest CP plasma levels and the rest of the cohort.

CP category	SCCPs	MCCPs	LCCPs
the participants with 10 highest CP plasma levels / the rest (median composition)			
Inhalation	7.2% / 6.0%	0.7% / 1.0%	0.3% / 0.4%
Dust ingestion	1.6% / 2.9%	1.6% / 3.3%	6.2% / 9.7%
Dietary intake	91% / 82%*	89% / 76%*	73% / 51%*
Dermal uptake	1.2% / 3.3%	6.7% / 14.1%	18% / 33%
the participants with 10 highest CP plasma levels / the rest (median intake, ng/kg BW/d)			
Inhalation	1.6 / 1.6	0.1 / 0.3	0.0 / 0.0
Dust ingestion	0.3 / 0.6	0.3 / 0.7	0.1 / 0.1
Dietary intake	26 / 16	23 / 15	0.6 / 0.5
Dermal uptake	0.5 / 0.7	1.9 / 2.9	0.3 / 0.3
the participants with 10 lowest CP plasma levels / the rest (median composition**)			
Inhalation	8.2% / 6.3%	0.7% / 1.0%	1.1% / 0.33%
Dust ingestion	6.9% / 2.0%	5.6% / 3.2%	17% / 8.6%
Dietary intake	81% / 89%	78% / 81%	32% / 66%*
Dermal uptake	3.5% / 2.9%	14% / 15%	39% / 25%
the participants with 10 lowest CP plasma levels / the rest (median intake, ng/kg BW/d)			
Inhalation	1.8 / 1.5	0.2 / 0.2	0.0 / 0.0
Dust ingestion	0.8 / 0.5	0.7 / 0.7	0.1 / 0.1
Dietary intake	14 / 20	12 / 16	0.3 / 0.6*
Dermal uptake	0.5 / 0.7	1.8 / 2.9	0.3 / 0.3

* $p < 0.05$; the intake dataset was log-transformed before statistical testing.

** the medians of the relative contributions of individual samples have been normalized; before normalization, the sum of median relative contributions was not equal to 100% due to that the median relative contributions of all the four exposure pathways were not from the same participant.

Table S11. Spearman rank correlations (r) between CP classes in plasma

	vSCCPs	SCCPs	MCCPs	LCCPs
vSCCPs		0.92*	0.81*	0.82*
SCCPs			0.80*	0.85*
MCCPs				0.75*
LCCPs				

* $p < 0.05$.

Table S12. Statistical comparisons between characteristics of the study group/diet type/residential environment based on questionnaire answers and median concentrations of CPs (with detection frequencies above 75%) in the diet (ng/g ww) and plasma samples (ng/g lipid).

	n	Median (Interquartile Range: IQR)			
		vSCCPs	SCCPs	MCCPs	LCCPs
**Diet (ng/g ww)**					
**Fish in the diet**					
Yes	37	–	4 (2.8-5)	7.9 (7.1-11)	0.83 (0.45-1.4)
No	22	–	4.2 (2.4-6)	8.6 (5.5-12)	0.59 (0.39-1.4)
% median difference [a]		–	10	9	34
**Egg in the diet**					
Yes	22	–	4 (3.1-5)	10 (7.2-13)	0.65 (0.39-1.3)
No	37	–	3.9 (2.7-6)	7.4 (6-11)	0.83 (0.45-1.6)
% median difference [a]		–	2	33*	24
**Meat in the diet**					
Yes	48	–	4 (3-6)	8.9 (6.1-12)	0.87 (0.44-1.6)
No	11	–	3 (2.6-5)	7.4 (7-8.8)	0.52 (0.39-0.94)
% median difference [a]		–	24	18	50
**Butter in the diet**					
Yes	25	–	5 (3.6-7)	10 (7.2-13)	0.91 (0.47-1.62)
No	34	–	3.4 (2.4-5)	7.3 (5.6-10)	0.71 (0.4-1.3)
% median difference [a]		–	34*	34	25
**Dairy product in the diet**					
Yes	56	–	4 (2.8-6)	8.2 (7-12)	0.79 (0.44-1.4)
No	3	–	1.7 (1.6-2)	5.5 (4.0-6.4)	0.41 (0.32-0.64)
% median difference [a]		–	81*	40	62
**Fruit in the diet**					
Yes	51	–	4 (2.5-5)	7.4 (6-11)	0.62 (0.40-1.4)
No	8	–	5 (4.1-6)	11 (9.5-13)	1.3 (0.86-2.0)
% median difference [a]		–	29	41*	72
**Gender of participants**					
Female	44	–	4 (3-5)	8 (6.7-11)	0.66 (0.43-1.4)
Male	15	–	5 (3-6)	7.9 (6.5-13)	1.0 (0.42-1.7)
% median difference [a]		–	29	1	43
**Plasma (ng/g lipid)**					
**Gender of participants**					
Female	44	–	2400 (1100-3700)	990 (<590-2300)	110 (<51-240)
Male	15	–	3000 (1100-5000)	1400 (780-4400)	130 (84-310)
% median difference [a]		–	22	36	17
**Working mainly in office/lab**					
Office	41	–	2300(1000-4500)	1000(<590-3400)	120(<51-260)
Laboratory	18	–	2500(1400-3600)	1200(770-1400)	110(54-250)
% median difference [a]		–	9	10	-11
**Owning a sofa**					
Yes	44	–	2900 (1400-4600)	1300 (770-4200)	130 (60-290)
No	15	–	1900 (<510-3500)	610 (<590-900)	40 (<51-200)
% median difference [a]		–	45*	73*	104
**Living room renovated**					
Yes	42	–	2100 (890-3500)	900(<590-1400)	110 (<51-260)
No	17	–	3500 (1800-4900)	1800 (1100-4300)	130 (97-250)
% median difference [a]		–	51	70*	22
**Fish consumption**					
Low (<median)	30	–	2000 (1000-4000)	1100 (<590-3600)	73 (<51-240)
High (>median)	29	–	3000 (1500-4000)	1100 (770-2100)	160 (79-280)
% median difference [a]		–	43	3	77

	n	Median (Interquartile Range: IQR)			
		**vSCCPs**	**SCCPs**	**MCCPs**	**LCCPs**
**Fat/oil consumption**					
Low	10	–	3100 (1000-4700)	1400 (770-3700)	150 (<51-310)
High	49	–	2100 (1200-3500)	950 (590-1800)	97 (51-240)
% median difference [a]		–	37	35	46
**Egg consumption**					
Low	29	–	2300 (1400-3500)	980 (620-2100)	110 (62-310)
High	30	–	2800 (780-4100)	1200 (<590-2700)	120 (<51-240)
% median difference [a]		–	19	22	12
**Diary product consumption**					
Low	29	–	2600 (760-4000)	1100 (<590-1800)	100 (<51-220)
High	30	–	2400 (1200-4600)	1100 (600-3400)	122 (<51-290)
% median difference [a]		–	10	1	18
**Butter consumption**					
Low	29	–	2400 (850-3500)	980 (<590-3700)	100 (<51-310)
High	30	–	2800 (1500-4400)	1300 (660-2600)	130 (<51-250)
% median difference [a]		–	16	29	22
**Meat consumption**					
Low	29	–	2200 (780-3600)	940 (<590-3800)	110 (<51-290)
High	30	–	3000 (1600-4100)	1200 (620-2600)	130 (65-250)
% median difference [a]		–	34	28	14
**Age of participants**					
20 – 29 years old	11	–	2600 (1400-4300)	1700 (910-3500)	130 (52-240)
30 – 39 years old	16	–	1800 (600-5400)	690 (<590-2700)	110 (<51-370)
40 – 49 years old	15	–	2000 (1300-2600)	930 (690-1300)	140 (57-290)
50 – years old	17	–	3400 (3000-4000)	1200 (1000-2400)	120 (<51-220)
Statistical significance [b]		–	$p>0.05$	$p>0.05$	$p>0.05$
**Living room floor material**					
parquet	41	–	2400 (1000-3800)	1000 (<590-2600)	100 (<51-220)
wood	8	–	1000 (550-3000)	690 (<590-3200)	51 (<51-190)
laminate	5	–	2800 (2500-3500)	1100 (800-1200)	220 (110-330)
flooring	3	–	7700 (4900-9100)	4300 (2500-7000)	360 (310-510)
Statistical significance [b]		–	$p>0.05$	$p>0.05$	$p<0.05$
**Residence house/apartment built**					
≤ 11 years (since 2002)	9	–	1800 (<510-3000)	950 (860-1700)	120 (52-210)
11 – 35 years	14	–	2500 (950-3900)	1100 (830-4000)	130 (<51-270)
35 – 50 years	16	–	3100 (2100-3500)	1200 (890-1600)	130 (59-230)
>50 years	17	–	1900 (600-4900)	770 (<590-1800)	110 (<51-310)
Statistical significance [b]		–	$p<0.05$	$p<0.05$	$p<0.05$
**Heating system**					
Central heating	8	–	3200 (1500-5200)	3500 (2100-4400)	180 (83-360)
Electric heating	21	–	3100 (1500-4700)	1300 (1000-4500)	170 (52-310)
Stove/fireplace	3	–	3500 (3200-4200)	2600 (1600-3500)	160 (100-210)
other	26	–	2400 (1200-4700)	790 (<590-1100)	110 (<51-210)
Statistical significance [b]		–	$p>0.05$	$p<0.05$	$p>0.05$

* $p<0.05$, Mann−Whitney U test;

[a] median difference in concentration of two categories in %, $((A - B)/((A + B)/2)) \times 100$;

[b] Kruskal-Wallis test.

Text S1: Sample collection

The sampling of diet, handwipes, stationary and personal air, dust and plasma is described in detail in Papadopoulou et al. (2016).[51] A short description for sampling is given here. Each participant received a balance and collected and weighed food portions of each meal and snack consumed over 2 consecutive days. Hand wipe samples were collected for assessing dermal exposure. The participants were advised to avoid hand washing for at least 60 min before the collection of the hand wipes. Each participant wiped the palm and the back from wrist to fingertips using two sterile gauze pads (3 in. × 3 in., Swift First Aid Inc., Valencia, CA), one for each hand. Two wipes were stored together as one sample. Stationary air was collected for 24 h using a low-volume active air pump (Leland Legacy, SKC Inc., Eighty Four, PA) with the sampling train containing two polyurethane foam (PUF) plugs and one glass fiber filter (GFF). The sampling volume was approximate 17 m^3 per sample. Personal air was collected for 24 h with a low-volume active air pump (SKC pump 224-PCMTX4, SKC Inc., Eighty Four, PA) with one sampling train containing two PUF plugs and a GFF. The sampling volume of air was c.a. 1.4 m^3 per participant. Thirteen personal air samples were available for the present CP study, as the other personal air samples went to studies of other indoor contaminants elsewhere. Settled dust was collected from all elevated surfaces at least 0.5 m above the floor such as tables, bookshelves, windowsills. A venous blood sample was collected from each participant by a research nurse at the NIPH during a scheduled appointment. Whole blood was collected in 10 mL plastic BD Vacutainer® whole blood tube with EDTA, to provide whole blood and plasma. Whole blood was centrifuged at 2200–2500 rpm and the plasma was transferred to a 10 mL tube.

The participants answered a questionnaire regarding the characteristics of their home, such as information on building and consumer goods. Each participant completed a questionnaire regarding their age, gender, weight and height, consumed food, eating habits, type and number of home appliances, and other characteristics of the indoor home environment.

Text S2: Sample Preparation

Plasma. Ten ng of internal standard $^{13}C_{10}$-1,5,5,6,6,10-hexachlorodecane ($^{13}C_{10}$-HCD, Cambridge Isotope Laboratories, Andover, MA) was added to 1 – 2 g plasma sample. The samples were liquid-liquid extracted according to the modified Jensen II extraction.[52, 53] The plasma was first mixed with 2-propanol with a v:v = 1:1, and then doubled the total volume by diluting with milli-Q water. The lipid was liquid-liquid extracted for 5 minutes using 6 mL 1:1 v:v *n*-hexane : methyl tert-butyl ether (MTBE). After centrifugation, the organic phase was collected, and the remaining phase was extracted using 3 mL 1:1 v:v *n*-hexane : MTBE. The organic phases were combined and then cleaned with a solution of potassium chloride (1%). The aqueous phase was re-extracted with *n*-hexane and the combined organic phases were evaporated to dryness using a gentle stream of nitrogen. The lipid weight was determined gravimetrically. Milli-Q water of 2 mL was used as a laboratory blank that was included prior to sample extraction in every batch of samples.

Diet. The diet samples of two consecutive days were first mixed based on the food portions, which comprised one diet sample for each participant. Diet samples were extracted according to Yuan et al.[7] The mixed diet samples were freeze-dried, and the water contents were determined gravimetrically after freeze-drying. A 2 – 3 g sample was spiked with $^{13}C_{10}$-HCD and extracted using accelerated solvent extraction (ASE 300; Dionex Europe, Leeds, UK; 100 °C and 1500 psi) and a mixture of 1:1 v:v n-hexane : dichloromethane (DCM) as solvent. The thermal equilibration time was 10 min, and the static extractions were performed within two cycles (8 min/cycle). The extract was gently dried using a nitrogen blow down apparatus, and the lipid content was determined gravimetrically. Pre-baked Na_2SO_4 (450 °C, 6 h) of 2 g was used as a laboratory blank that was included prior to sample extraction in every batch of samples.

Column clean-up. The lipid extracts of plasma or diet samples were cleaned-up on a multilayer SPE column containing, from bottom to top, 2 g deactivated silica (2.5% H_2O w/w), 8 g acid silica (44% condensed sulfuric acid, w/w), and 4 g anhydrous sodium sulfate.[54] The eluent of 1:1 v:v n-hexane : DCM was collected and reconstituted in DCM with 20 ng of Dechlorane-603 (Occidental Chemical Corp.) prior to instrumental analysis. All solvents and reagents were of highest commercial purity.

Text S3: MS settings and CP quantification

UPLC-APCI-Orbitrap-HRMS (Q Exactive, Thermo Fisher Scientific, San Jose, USA) was operated in full-scan mode (m/z 250–2000) with a resolution of 120 000 FWHM. The instrumental settings were optimized using a SCCP mixture (51.5% Cl, 15 ng/μL) and a LCCP mixture (Witaclor 549, 49% Cl, 5 ng/μL) as follows: DCM flow rate 0.028 mL/min, capillary temperature 250 °C, Aux (auxiliary) gas heater temperature 250 °C, spray current 5.7 μA, maximum IT (ion time) 250 ms, AGC (automatic gain control) target 5e6, sheath gas flow rate 17 arbs, and Aux gas flow rate 1 arb.

A schematic of the quantification procedure was given in Du et al.[55] Briefly, SCCPs, MCCPs, and LCCPs were quantified using a pattern-deconvolution algorithm developed by Bogdal et al.[56] The $C_{\geqslant 10}$ homologue profile of each sample was reconstructed as a linear combination of 20 CP products (Table S2). Then, the instrumental response factors of SCCPs, MCCPs, and LCCPs in the sample were calculated, according to relative contributions of respective CP products. vSCCPs in the sample were quantified using CPs from a local retailer (CP-52) as the reference standard. The response factor of vSCCPs was calculated from $C_{6–9}$ components of CP-52 (which are 0.94 ± 0.30% w/w as given in Zhou et al.[57]). Individual homologues (C_nCl_m) and chlorine content (%Cl, w/w) were semi-quantified based on their relative instrumental signals. For %Cl of a sample:

$$\%\mathrm{Cl} = \frac{\sum(\%\mathrm{Cl}_{\mathrm{C}_n\mathrm{Cl}_m} \times \text{relative instrumental signal}_{\mathrm{C}_n\mathrm{Cl}_m})}{\sum \text{relative instrumental signal}_{\mathrm{C}_n\mathrm{Cl}_m}} \qquad \text{(S1)}$$

where $\%Cl_{C_nCl_m}$ is the chlorine content of C_nCl_m and calculated according to the chemical formula $C_nH_{2n+2-m}Cl_m$.

Text S4: Total exposure calculation from external media

Inhalation and dust ingestion exposure calculation. The calculation was according to the U.S. EPA *Exposure Factors Handbook*:[58]

$$\text{exposure}_{\text{inhalation}} = \frac{C_{\text{air}} \times \text{IR} \times \text{ED}}{\text{BW}} \quad \text{(S2)}$$

$$\text{exposure}_{\text{ingestion}} = \frac{C_{\text{dust}} \times \text{DI}}{\text{BW}} \quad \text{(S3)}$$

where C_{air} and C_{dust} are the concentrations of CPs in air (ng/m^3) and dust (ng/g), respectively. IR is the inhalation rate (m^3/day) which was assigned on the basis of body weight (BW, range: 52 – 125 kg), gender, and age of each participant according to the U.S. EPA *Exposure Factors Handbook*.[58] ED is the exposure duration as a time fraction of the 24 h. Using stationary air, the ED is calculated based on average hours spent indoors per day (range: 18 – 23.8 h) as assessed by questionnaires, which ranges from 0.75 to 0.99. Using personal air, the ED is 24 h/24 h = 1. DI is the mean daily dust intake (30 mg/d for adults).

Dermal exposure calculation. Dermal exposure to CPs via hand contact (ng/kg bw/d) was then estimated for each participant:

$$\text{exposure}_{\text{dermal}} = \frac{C_{\text{hw}} \times \text{SA} \times \text{ED} \times \text{EF}}{\text{BW}} \quad \text{(S4)}$$

where C_{hw} is the surface-area normalized mass of CPs in hand wipes (ng/cm^2), SA is hand skin surface area (cm^2) estimated according to U.S. EPA *Exposure Factors Handbook*.[58] ED is the exposure duration of 24 h (t/24, where t is assumed to be 24 h), EF is the exposure frequency which is assumed to be 1 event/day.

Relative contribution of an exposure pathway to total external exposure. The relative contribution (%) of an exposure pathway *i* is calculated based on the share of $\text{exposure}_i \times AF_{CPs,\,i}$ in the total daily exposure $[\Sigma(\text{exposure}_i \times AF_{CPs,\,i})]$ (ng/kg BW/d):

$$\text{Relative contribution} = \frac{\text{exposure}_i \times AF_{CPs,i}}{\Sigma(\text{exposure}_i \times AF_{CPs,i})} \times 100\% \quad \text{(S5)}$$

where *i* consist of dietary, inhalation, (dust) ingestion, and dermal exposure pathways. $AF_{CPs,\,i}$ is the absorption fraction of exposure pathway *i*, which is estimated based on the $\log K_{OW}$ and/or the bioaccessibility of each CP class (Table S4) except for $AF_{CPs,\,inhalation}$ which is assumed to be 100% bioavailable.[15, 59]

Text S5: Calculation of Body Lipid (BL)

BL is the body lipid mass estimated from each individual participant's height and body weight (g) using *eq* S1.

$$\text{Adult body fat (\%)} = (1.20\times\text{BMI}) + (0.23\times\text{Age}) - (10.8\times\text{sex}) - 5.4 \quad \text{(S6)}$$

where body mass index (BMI) for each participant was calculated based on height and weight provided during questionnaires, age was from the questionnaires, sex is 1 for males and 0 for females, the source of the Body Fat Percentage Formula is BMI Calories (http://bmi-calories.com/body-fat-percentage-calculator.html).

Text S6: Forensic Fingerprinting

The approach was adopted from a previous study[7] with additional consideration of bioaccessibility of CP homologues. The C_nCl_m-profiles of SCCPs, MCCPs, and LCCPs of each plasma sample were respectively linearly superimposed[56] using the bioaccessibility-calibrated profiles of the corresponding four external exposure media using *eq*. S7.

$$\begin{pmatrix} \text{RA}_{1,\text{plasma}} \\ \text{RA}_{2,\text{plasma}} \\ \cdots \\ \text{RA}_{i,\text{plasma}} \end{pmatrix} = \begin{pmatrix} \text{cRA}_{1,1} & \text{cRA}_{1,2} & \cdots & \text{cRA}_{1,j} \\ \text{cRA}_{2,1} & \text{cRA}_{2,2} & \cdots & \text{cRA}_{2,j} \\ \cdots & \cdots & \cdots & \cdots \\ \text{cRA}_{i,1} & \text{cRA}_{i,2} & \cdots & \text{cRA}_{i,j} \end{pmatrix} \cdot \begin{pmatrix} x_1 \\ x_2 \\ \cdots \\ x_j \end{pmatrix} \quad \text{(S7)}$$

where $\text{RA}_{i,\text{plamsa}}$ is the relative instrumental response area of homologue *i* in the plasma sample, $\text{cRA}_{i,j}$ is the bioaccessibility-calibrated instrumental response area of homologue *i* in external exposure medium *j*, and x_j is the percentage contribution of the external exposure medium *j* in the linear-combined pattern of the plasma sample. The instrumental response area of each homologue *i* in external exposure medium *j* ($\text{RA}_{i,j}$) is calibrated using *eq* S8:

$$\text{cRA}_{i,j} = \text{RA}_{i,j} \times \text{BA}_{i,j} \quad \text{(S8)}$$

where $\text{BA}_{i,j}$ is the bioaccessibility of each homologue *i* in external exposure medium *j* which was calculated based in a piecewise-defined function *eq* S9

$$\text{BA}_{i,j} = \begin{cases} \text{BA}_{i,\text{air}} = 100\% \\ \text{BA}_{i,\text{other}} \end{cases} \quad \text{(S9)}$$

The bioaccessibility of each homologue *i* in external exposure media other than air $\text{BA}_{i,\text{other}}$ was calculated using *eq* S10 reported by Du et al.[19]:

$$\text{BA}_{i,\text{other}} = 0.057 \times \text{carbon number} - 0.994 \times \text{LN(chlorine number)} + 3.073 \quad \text{(S10)}$$

The *eq* S5 was built up based on $C_{10\text{-}17}$ and $Cl_{5\text{-}10}$. For homologues out of such range, i.e., if $\text{BA}_{i,\text{other}}$ >100%, the $\text{BA}_{i,\text{other}}$ is reassigned 100%; if $\text{BA}_{i,\text{other}}$ <8%, the $\text{BA}_{i,\text{other}}$ is reassigned 7.9%.

The superimposition is optimized for each participant with the goal of maximizing the goodness-of-fit R^2 between the plasma profile and the superimposed one. R^2 ranges between 0 and 1, and $R^2 = 1$ means a perfect superimposition. R^2 was calculated by the following equation[60]:

$$R^2 = \frac{(\sum \text{RA}_{i,\text{pattern\_a}} \cdot \text{RA}_{i,\text{pattern\_b}} - \frac{1}{i}\sum \text{RA}_{i,\text{pattern\_a}} \cdot \sum \text{RA}_{i,\text{pattern\_b}})^2}{(\sum \text{RA}_{i,\text{pattern\_a}}{}^2 - \frac{1}{i}(\sum \text{RA}_{i,\text{pattern\_a}})^2) \cdot (\sum \text{RA}_{i,\text{pattern\_b}}{}^2 - \frac{1}{i}(\sum \text{RA}_{i,\text{pattern\_b}})^2)} \quad \text{(S11)}$$

where $\text{RA}_{i,\text{pattern-a}}$ and $\text{RA}_{i,\text{pattern-b}}$ are the relative instrumental response area of homologue *i* in the compared two homologue profiles, respectively. An example of a forensic fingerprint is shown in Figure S1.

Text S7: Determination of Chlorinated Paraffin in Human Serum

In a pilot experiment, the CP concentration in one human serum sample was approximately 70 ng/g ww. Based on this, the extraction and clean-up method for CP analysis in human blood samples was validated using a spike-and-recovery test. Human serum was purchased from Karolinska Institutet, Stockholm, Sweden. Ten aliquots of 2-gram serum samples were used for method validation. An incubation test was implemented to demonstrate that the method was capable of sufficiently extracting CPs in serum. Five aliquots were incubated with a known amount (158 ng) of CP standard mixtures, consisting of SCCPs, MCCPs, and LCCPs. After the addition of the standard mixture, the serum was stored at 4°C overnight as given by Richthoff et al.[61] Then CPs were analyzed in both the unspiked and spiked serum samples.

The mean total CP concentration in the unspiked serum sample was 98.5 ± 21.3 ng/g ww, or 11400 ± 2140 ng/g lipid (n = 5). The CP homologue profile is shown in Figure S1, in which vSCCPs (C_9) contributed 2% of the total CPs. The theoretical concentration of total CPs in the incubated spiked serum sample was 186 ng/g (sum of the native CPs in serum and the CPs added), and the measured concentration was 183 ± 13 ng/g (n = 5), which shows that the method extracted 98% ± 7% of CPs from the serum.

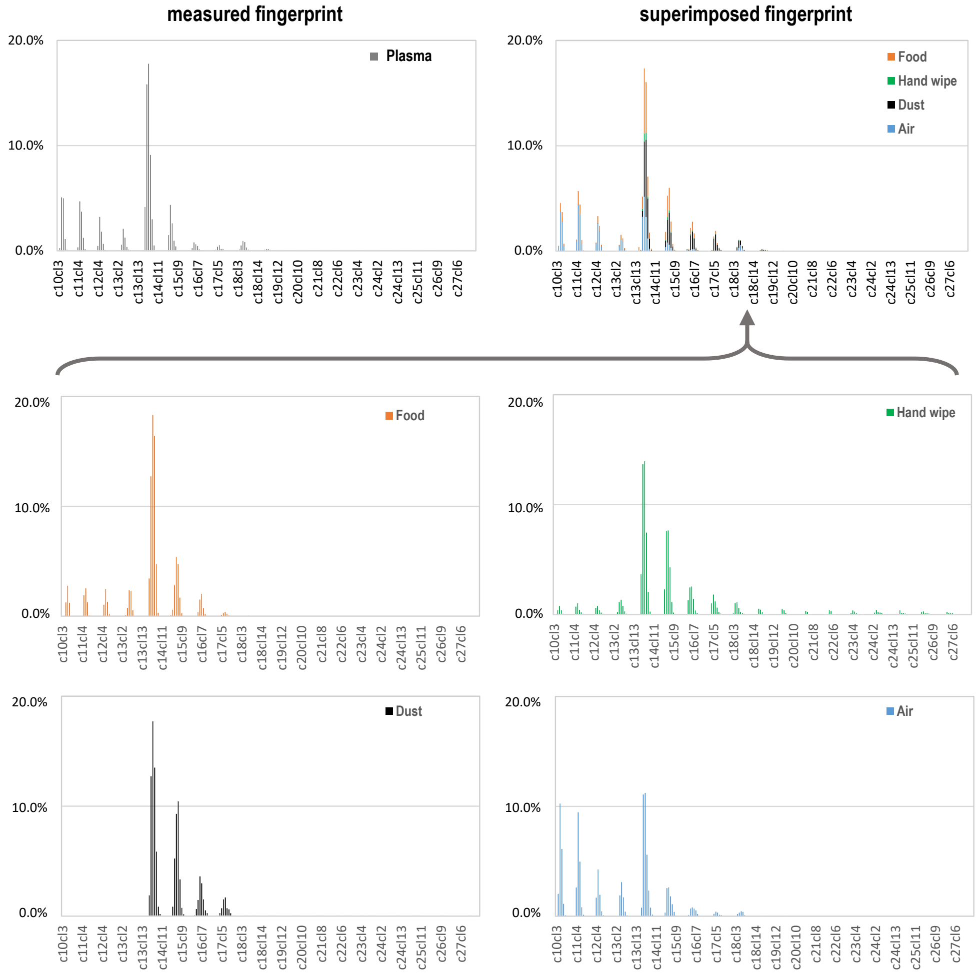

Figure S1. Forensic fingerprinting CP homologues in a plasma sample (R^2 = 0.94). Forensic fingerprinting CP homologues in a plasma sample (R^2 = 0.94). The vertical axis is the relative abundance of C_nCl_m, and the sum of all C_nCl_m signals equals 1. The horizontal axis lists the C_nCl_m. The fingerprinting starts from C_{10} due to the low detection frequencies of vSCCPs in multiple matrices.

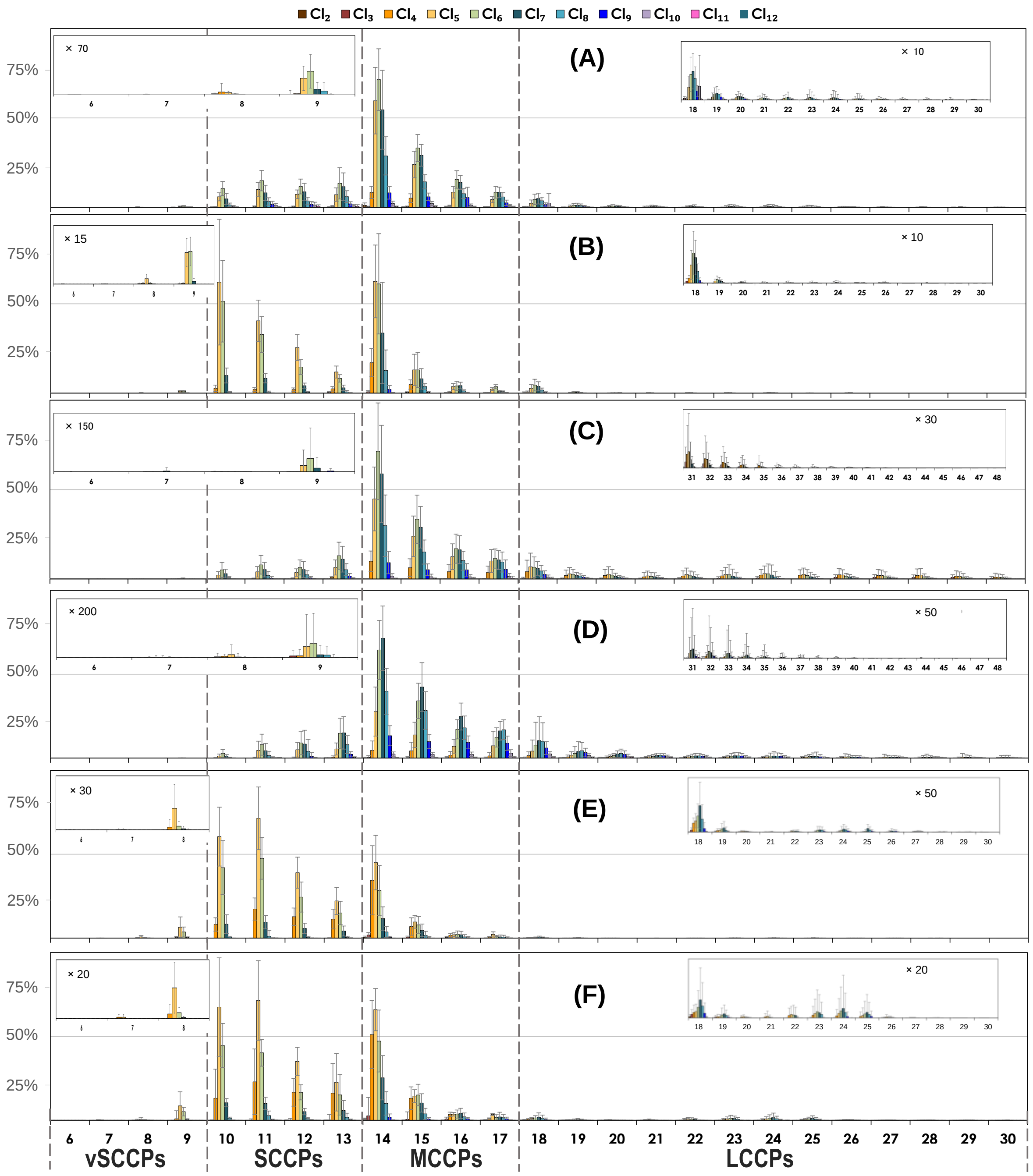

Figure S2. Mean relative abundance of CP homologues with standard deviation error bars for (A) diet (this study), (B) plasma (this study), (C) hand wipe,[14] (D) settled dust,[15] (E) stationary air,[15] and (F) personal air samples[15] of the Norwegian cohort. The horizontal axes represent carbon chain length.

References

(1) Sprengel, J.; Wieselmann, S.; Kröpfl, A.; Vetter, W. High amounts of chlorinated paraffins in oil-based vitamin E dietary supplements on the German market. *Environment International* **2019**, *128*, 438-445. DOI: https://doi.org/10.1016/j.envint.2019.04.065.

(2) Dong, S.; Li, X.; Su, X.; Wang, P. Concentrations and congener group profiles of short- and medium-chain chlorinated paraffins in animal feed materials. *Science of The Total Environment* **2019**, *647*, 676-681. DOI: https://doi.org/10.1016/j.scitotenv.2018.08.017.

(3) Castro, M.; Sobek, A.; Yuan, B.; Breitholtz, M. Bioaccumulation Potential of CPs in Aquatic Organisms: Uptake and Depuration in Daphnia magna. *Environmental Science & Technology* **2019**, *53* (16), 9533-9541. DOI: 10.1021/acs.est.9b01751.

(4) Yuan, B.; Fu, J.; Wang, Y.; Jiang, G. Short-chain chlorinated paraffins in soil, paddy seeds (Oryza sativa) and snails (Ampullariidae) in an e-waste dismantling area in China: Homologue group pattern, spatial distribution and risk assessment. *Environmental Pollution* **2017**, *220*, 608-615.

(5) Chen, W.; Hou, X.; Liu, Y.; Hu, X.; Liu, J.; Schnoor, J. L.; Jiang, G. Medium- and Short-Chain Chlorinated Paraffins in Mature Maize Plants and Corresponding Agricultural Soils. *Environmental Science & Technology* **2021**, *55* (8), 4669-4678. DOI: 10.1021/acs.est.0c05111.

(6) Gallistl, C.; Sprengel, J.; Vetter, W. High levels of medium-chain chlorinated paraffins and polybrominated diphenyl ethers on the inside of several household baking oven doors. *Sci. Total Environ.* **2018**, *615*, 1019-1027. DOI: https://doi.org/10.1016/j.scitotenv.2017.09.112.

(7) Yuan, B.; Strid, A.; Darnerud, P. O.; de Wit, C. A.; Nyström, J.; Bergman, Å. Chlorinated paraffins leaking from hand blenders can lead to significant human exposures. *Environment International* **2017**, *109*, 73-80. DOI: https://doi.org/10.1016/j.envint.2017.09.014.

(8) Wang, C.; Gao, W.; Liang, Y.; Jiang, Y.; Wang, Y.; Zhang, Q.; Jiang, G. Migration of chlorinated paraffins from plastic food packaging into food simulants: Concentrations and differences in congener profiles. *Chemosphere* **2019**, *225*, 557-564. DOI: https://doi.org/10.1016/j.chemosphere.2019.03.039.

(9) Gao, W.; Lin, Y.; Liang, Y.; Wang, Y.; Jiang, L.; Wang, Y.; Jiang, G. Percutaneous penetration and dermal exposure risk assessment of chlorinated paraffins. *Journal of Hazardous Materials* **2021**, *416*, 126178. DOI: https://doi.org/10.1016/j.jhazmat.2021.126178.

(10) Friden, U. E.; McLachlan, M. S.; Berger, U. Chlorinated paraffins in indoor air and dust: concentrations, congener patterns, and human exposure. *Environ. Int.* **2011**, *37* (7), 1169-1174. DOI: 10.1016/j.envint.2011.04.002.

(11) Xu, J.; Gao, Y.; Zhang, H.; Zhan, F.; Chen, J. Dispersion of Short- and Medium-Chain Chlorinated Paraffins (CPs) from a CP Production Plant to the Surrounding Surface Soils and Coniferous Leaves. *Environ. Sci. Technol.* **2016**, *50* (23), 12759-12766. DOI: 10.1021/acs.est.6b03595.

(12) Chen, H.; Lam, J. C. W.; Zhu, M.; Wang, F.; Zhou, W.; Du, B.; Zeng, L.; Zeng, E. Y. Combined Effects of Dust and Dietary Exposure of Occupational Workers and Local Residents to Short- and Medium-Chain Chlorinated Paraffins in a Mega E-Waste Recycling Industrial Park in South China. *Environmental Science & Technology* **2018**, *52* (20), 11510-11519. DOI: 10.1021/acs.est.8b02625.

(13) Zeng, L.; Wang, T.; Han, W.; Yuan, B.; Liu, Q.; Wang, Y.; Jiang, G. Spatial and Vertical Distribution of Short Chain Chlorinated Paraffins in Soils from Wastewater Irrigated Farmlands. *Environmental Science & Technology* **2011**, *45* (6), 2100-2106. DOI: 10.1021/es103740v.

(14) Yuan, B.; Tay, J. H.; Papadopoulou, E.; Haug, L. S.; Padilla-Sánchez, J. A.; de Wit, C. A. Complex Mixtures of Chlorinated Paraffins Found in Hand Wipes of a Norwegian Cohort. *Environ. Sci. Technol. Lett.* **2020**, *7* (3), 198-205. DOI: 10.1021/acs.estlett.0c00090.

(15) Yuan, B.; Tay, J. H.; Padilla-Sánchez, J. A.; Papadopoulou, E.; Haug, L. S.; de Wit, C. A. Human Exposure to Chlorinated Paraffins via Inhalation and Dust Ingestion in a Norwegian Cohort. *Environ. Sci. Technol.* **2021**, *55* (2), 1145-1154. DOI: 10.1021/acs.est.0c05891.

(16) Xia, D.; Gao, L.; Zheng, M.; Sun, Y.; Qiao, L.; Huang, H.; Zhang, H.; Fu, J.; Wu, Y.; Li, J.; et al. Identification and evaluation of chlorinated nonane paraffins in the environment: A persistent organic pollutant candidate for the Stockholm Convention? *J. Hazard. Mater.* **2019**, *371*, 449-455. DOI: https://doi.org/10.1016/j.jhazmat.2019.02.089.

(17) Hilger, B.; Fromme, H.; Volkel, W.; Coelhan, M. Effects of Chain Length, Chlorination Degree, and Structure on the Octanol-Water Partition Coefficients of Polychlorinated n-Alkanes. *Environ. Sci. Technol.* **2011**, *45* (7), 2842-2849. DOI: 10.1021/es103098b.

(18) Muir, D.; Stern, G.; Tomy, G. Chlorinated Paraffins. In *Volume 3 Anthropogenic Compounds Part K*, Hutzinger, O., Paasivirta, J. Eds.; Springer Berlin Heidelberg, 2000; pp 203-236.

(19) Du, X.; Zhou, Y.; Li, J.; Wu, Y.; Zheng, Z.; Yin, G.; Qiu, Y.; Zhao, J.; Yuan, G. Evaluating oral and inhalation bioaccessibility of indoor dust-borne short- and median-chain chlorinated paraffins using in vitro Tenax-assisted physiologically based method. *J. Hazard. Mater.* **2021**, *402*, 123449. DOI: https://doi.org/10.1016/j.jhazmat.2020.123449.

(20) Dong, Z.; Li, T.; Wan, Y.; Sun, Y.; Hu, J. Physiologically Based Pharmacokinetic Modeling for Chlorinated Paraffins in Rats and Humans: Importance of Biliary Excretion. *Environmental Science & Technology* **2020**, *54* (2), 938-946. DOI: 10.1021/acs.est.9b03991.

(21) Braekevelt, E.; Tittlemier, S. A.; Tomy, G. T. Direct measurement of octanol–water partition coefficients of some environmentally relevant brominated diphenyl ether congeners. *Chemosphere* **2003**, *51* (7), 563-567. DOI: https://doi.org/10.1016/S0045-6535(02)00841-X.

(22) Krätschmer, K.; Schächtele, A.; Vetter, W. Short- and medium-chain chlorinated paraffin exposure in South Germany: A total diet, meal and market basket study. *Environmental Pollution* **2021**, *272*, 116019. DOI: https://doi.org/10.1016/j.envpol.2020.116019.

(23) Krätschmer, K.; Schächtele, A.; Malisch, R.; Vetter, W. Chlorinated paraffins (CPs) in salmon sold in southern Germany: Concentrations, homologue patterns and relation to other persistent organic pollutants. *Chemosphere* **2019**, *227*, 630-637. DOI: https://doi.org/10.1016/j.chemosphere.2019.04.016.

(24) Mézière, M. Analytical strategy development for the analysis of chlorinated paraffins: Study of the fate of those contaminants of emerging concern in the laying hens and contribution of the evaluation of the human dietary exposure. Nantes, Ecole nationale vétérinaire, 2020.

(25) Iino, F.; Takasuga, T.; Senthilkumar, K.; Nakamura, N.; Nakanishi, J. Risk assessment of short-chain chlorinated paraffins in Japan based on the first market basket study and species sensitivity distributions. *Environmental science & technology* **2005**, *39* (3), 859-866.

(26) Harada, K. H.; Takasuga, T.; Hitomi, T.; Wang, P.; Matsukami, H.; Koizumi, A. Dietary exposure to short-chain chlorinated paraffins has increased in Beijing, China. *Environ Sci Technol* **2011**, *45* (16), 7019-7027. DOI: 10.1021/es200576d.

(27) Lee, S.; Choo, G.; Ekpe, O. D.; Kim, J.; Oh, J.-E. Short-chain chlorinated paraffins in various foods from Republic of Korea: Levels, congener patterns, and human dietary exposure. *Environmental Pollution* **2020**, *263*, 114520. DOI: https://doi.org/10.1016/j.envpol.2020.114520.

(28) Li, H.; Gao, S.; Yang, M.; Zhang, F.; Cao, L.; Xie, H.; Chen, X.; Cai, Z. Dietary exposure and risk assessment of short-chain chlorinated paraffins in supermarket fresh products in Jinan, China. *Chemosphere* **2020**, *244*, 125393. DOI: https://doi.org/10.1016/j.chemosphere.2019.125393.

(29) Gao, W.; Cao, D.; Wang, Y.; Wu, J.; Wang, Y.; Wang, Y.; Jiang, G. External Exposure to Short- and Medium-Chain Chlorinated Paraffins for the General Population in Beijing, China. *Environ. Sci. Technol.* **2018**, *52* (1), 32-39. DOI: 10.1021/acs.est.7b04657.

(30) Huang, H.; Gao, L.; Zheng, M.; Li, J.; Zhang, L.; Wu, Y.; Wang, R.; Xia, D.; Qiao, L.; Cui, L.; et al. Dietary exposure to short- and medium-chain chlorinated paraffins in meat and meat products from 20 provinces of China. *Environmental Pollution* **2018**, *233*, 439-445. DOI: https://doi.org/10.1016/j.envpol.2017.10.022.

(31) Zeng, Y.; Huang, C.; Luo, X.; Liu, Y.; Ren, Z.; Mai, B. Polychlorinated biphenyls and chlorinated paraffins in home-produced eggs from an e-waste polluted area in South China: Occurrence and human dietary exposure. *Environment International* **2018**, *116*, 52-59. DOI: https://doi.org/10.1016/j.envint.2018.04.006.

(32) Cui, L.; Gao, L.; Zheng, M.; Li, J.; Zhang, L.; Wu, Y.; Qiao, L.; Xu, C.; Wang, K.; Huang, D. Short- and Medium-Chain Chlorinated Paraffins in Foods from the Sixth Chinese Total Diet Study: Occurrences and Estimates of Dietary Intakes in South China. *J Agr Food Chem* **2020**, *68* (34), 9043-9051. DOI: 10.1021/acs.jafc.0c03491.

(33) Bergman, Å.; Yuan, B.; Bignert, A.; Andersson, P.; West, C.; Domellöf, M. *Analys av klorparaffiner i bröstmjölk och serum*; Stockholm, 2022. http://urn.kb.se/resolve?urn=urn:nbn:se:naturvardsverket:diva-9961 (accessed 2022-02-09).

(34) van Mourik, L. M.; Toms, L.-M. L.; He, C.; Banks, A.; Hobson, P.; Leonards, P. E. G.; de Boer, J.; Mueller, J. F. Evaluating age and temporal trends of chlorinated paraffins in pooled serum collected from males in Australia between 2004 and 2015. *Chemosphere* **2020**, *244*, 125574. DOI: https://doi.org/10.1016/j.chemosphere.2019.125574.

(35) Qiao, L.; Gao, L.; Zheng, M.; Xia, D.; Li, J.; Zhang, L.; Wu, Y.; Wang, R.; Cui, L.; Xu, C. Mass Fractions, Congener Group Patterns, and Placental Transfer of Short-and Medium-Chain Chlorinated Paraffins in Paired Maternal and Cord Serum. *Environmental science & technology* **2018**, *52* (17), 10097-10103.

(36) Li, T.; Wan, Y.; Gao, S.; Wang, B.; Hu, J. High-Throughput Determination and Characterization of Short-, Medium-, and Long-Chain Chlorinated Paraffins in Human Blood. *Environ. Sci. Technol.* **2017**, *51* (6), 3346-3354.

(37) Chen, H.; Zhou, W.; Lam, J. C. W.; Ge, J.; Li, J.; Zeng, L. Blood partitioning and whole-blood-based maternal transfer assessment of chlorinated paraffins in mother-infant pairs from South China. *Environment International* **2020**, *142*, 105871. DOI: https://doi.org/10.1016/j.envint.2020.105871.

(38) Aamir, M.; Yin, S.; Guo, F.; Liu, K.; Xu, C.; Liu, W. Congener-Specific Mother–Fetus Distribution, Placental Retention, and Transport of C10–13 and C14–17 Chlorinated Paraffins in Pregnant Women. *Environmental Science & Technology* **2019**, *53* (19), 11458-11466. DOI: 10.1021/acs.est.9b02116.

(39) Liu, Y.; Aamir, M.; Li, M.; Liu, K.; Hu, Y.; Liu, N.; Xu, Y.; Du, J.; Xu, J.; Liu, W. Prenatal and postnatal exposure risk assessment of chlorinated paraffins in mothers and neonates: Occurrence, congener profile, and transfer behavior. *Journal of Hazardous Materials* **2020**, *395*, 122660. DOI: https://doi.org/10.1016/j.jhazmat.2020.122660.

(40) Zhao, N.; Fang, X.; Zhang, S.; Zhu, Y.; Ding, L.; Xu, C. Male renal functions are associated with serum short- and medium-chain chlorinated paraffins in residents from Jinan, China. *Environment International* **2021**, *153*, 106514. DOI: https://doi.org/10.1016/j.envint.2021.106514.

(41) Xu, J.; Guo, W.; Wei, L.; Gao, Y.; Zhang, H.; Zhang, Y.; Sun, M.; Chen, J. Validation of a HRGC–ECNI/LRMS method to monitor short-chain chlorinated paraffins in human plasma. *J Environ Sci-China* **2019**, *75*, 289-295. DOI: https://doi.org/10.1016/j.jes.2018.04.004.

(42) Zhou, Y.; Yuan, B.; Nyberg, E.; Yin, G.; Bignert, A.; Glynn, A.; Odland, J. Ø.; Qiu, Y.; Sun, Y.; Wu, Y.; et al. Chlorinated Paraffins in Human Milk from Urban Sites in China, Sweden, and Norway. *Environ. Sci. Technol.* **2020**, *54* (7), 4356-4366. DOI: 10.1021/acs.est.9b06089.

(43) Tomy, G. T. The mass spectrometric characterization of polychlorinated n-alkanes and the methodology for their analysis in the environment. Dissertation University of Manitoba, 1997. http://hdl.handle.net/1993/745.

(44) Thomas, G. O.; Farrar, D.; Braekevelt, E.; Stern, G.; Kalantzi, O. I.; Martin, F. L.; Jones, K. C. Short and medium chain length chlorinated paraffins in UK human milk fat. *Environ Int* **2006**, *32* (1), 34-40. DOI: 10.1016/j.envint.2005.04.006.

(45) Cao, Y.; Harada, K. H.; Hitomi, T.; Niisoe, T.; Wang, P.; Shi, Y.; Yang, H.-R.; Takasuga, T.; Koizumi, A. Lactational exposure to short-chain chlorinated paraffins in China, Korea, and Japan. *Chemosphere* **2017**, *173*, 43-48. DOI: https://doi.org/10.1016/j.chemosphere.2016.12.078.

(46) Krätschmer, K.; Malisch, R.; Vetter, W. Chlorinated Paraffin Levels in Relation to Other Persistent Organic Pollutants Found in Pooled Human Milk Samples from Primiparous Mothers in 53 Countries. *Environ Health Persp* **2021**, *129* (8), 087004. DOI: doi:10.1289/EHP7696.

(47) Xia, D.; Gao, L.; Zheng, M.; Li, J.; Zhang, L.; Wu, Y.; Tian, Q.; Huang, H.; Qiao, L. Human Exposure to Short- and Medium-Chain Chlorinated Paraffins via Mothers' Milk in Chinese Urban Population. *Environmental science & technology* **2017**, *51* (1), 608-615. DOI: 10.1021/acs.est.6b04246.

(48) Xia, D.; Gao, L. R.; Zheng, M. H.; Li, J. G.; Zhang, L.; Wu, Y. N.; Qiao, L.; Tian, Q. C.; Huang, H. T.; Liu, W. B.; et al. Health risks posed to infants in rural China by exposure to short- and medium-chain chlorinated paraffins in breast milk. *Environ Int* **2017**, *103*, 1-7. DOI: 10.1016/j.envint.2017.03.013.

(49) Xu, C.; Wang, K.; Gao, L.; Zheng, M.; Li, J.; Zhang, L.; Wu, Y.; Qiao, L.; Huang, D.; Wang, S.; et al. Highly elevated levels, infant dietary exposure and health risks of medium-chain chlorinated paraffins in breast milk from China: Comparison with short-chain chlorinated paraffins. *Environmental Pollution* **2021**, *279*, 116922. DOI: https://doi.org/10.1016/j.envpol.2021.116922.

(50) Martínez, C.; Martínez Arroyo, A.; Barrientos Alemán, D.; Gavilán García, A.; Caba, M.; Calderón Garcidueñas, A. L.; Mora, A.; Zenteno, E. Persistent organic compounds in human milk and evaluation of the effectiveness of the Stockholm convention in Mexico. *Environmental Advances* **2022**, *8*, 100190. DOI: https://doi.org/10.1016/j.envadv.2022.100190.

(51) Papadopoulou, E.; Padilla-Sanchez, J. A.; Collins, C. D.; Cousins, I. T.; Covaci, A.; de Wit, C. A.; Leonards, P. E. G.; Voorspoels, S.; Thomsen, C.; Harrad, S.; et al. Sampling strategy for estimating human exposure pathways to consumer chemicals. *Emerg. Contam.* **2016**, *2* (1), 26-36. DOI: https://doi.org/10.1016/j.emcon.2015.12.002.

(52) Jensen, S.; Häggberg, L.; Jörundsdóttir, H.; Odham, G. A Quantitative Lipid Extraction Method for Residue Analysis of Fish Involving Nonhalogenated Solvents. *J Agr Food Chem* **2003**, *51* (19), 5607-5611. DOI: 10.1021/jf0301201.

(53) Sahlström, L. M. O.; Sellström, U.; de Wit, C. A.; Lignell, S.; Darnerud, P. O. Feasibility Study of Feces for Noninvasive Biomonitoring of Brominated Flame Retardants in Toddlers. *Environmental Science & Technology* **2015**, *49* (1), 606-615. DOI: 10.1021/es504708c.

(54) Yuan, B.; Benskin, J. P.; Chen, C.-E. L.; Bergman, Å. Determination of Chlorinated Paraffins by Bromide-Anion Attachment Atmospheric-Pressure Chemical Ionization Mass Spectrometry. *Environ. Sci. Technol. Lett.* **2018**, *5* (6), 348-353. DOI: 10.1021/acs.estlett.8b00216.

(55) Du, X.; Yuan, B.; Zhou, Y.; de Wit, C. A.; Zheng, Z.; Yin, G. Chlorinated Paraffins in Two Snake Species from the Yangtze River Delta: Tissue Distribution and Biomagnification. *Environ. Sci. Technol.* **2020**, *54* (5), 2753-2762. DOI: 10.1021/acs.est.9b06467.

(56) Bogdal, C.; Alsberg, T.; Diefenbacher, P. S.; MacLeod, M.; Berger, U. Fast quantification of chlorinated paraffins in environmental samples by direct injection high-resolution mass spectrometry with pattern deconvolution. *Anal. Chem.* **2015**, *87* (5), 2852-2860. DOI: 10.1021/ac504444d.

(57) Zhou, Y.; de Wit, C. A.; Yin, G.; Du, X.; Yuan, B. Shorter than short-chain: Very short-chain chlorinated paraffins (vSCCPs) found in wildlife from the Yangtze River Delta. *Environ. Int.* **2019**, *130*, 104955. DOI: https://doi.org/10.1016/j.envint.2019.104955.

(58) USEPA. Exposure Factors Handbook; EPA/600/R-09/052F. **2011**.

(59) Tay, J. H.; Sellstrom, U.; Papadopoulou, E.; Padilla-Sanchez, J. A.; Haug, L. S.; de Wit, C. A. Human Exposure to Legacy and Emerging Halogenated Flame Retardants via Inhalation and Dust Ingestion in a Norwegian Cohort. *Environ. Sci. Technol.* **2017**, *51* (14), 8176-8184. DOI: 10.1021/acs.est.7b02114.

(60) Yuan, B.; Bogdal, C.; Berger, U.; MacLeod, M.; Gebbink, W. A.; Alsberg, T.; de Wit, C. A. Quantifying Short-Chain Chlorinated Paraffin Congener Groups. *Environ. Sci. Technol.* **2017**, *51* (18), 10633-10641. DOI: 10.1021/acs.est.7b02269.

(61) Richthoff, J.; Rylander, L.; Jönsson, B. A.; Akesson, H.; Hagmar, L.; Nilsson-Ehle, P.; Stridsberg, M.; Giwercman, A. Serum levels of 2,2',4,4',5,5'-hexachlorobiphenyl (CB-153) in relation to markers of reproductive function in young males from the general Swedish population. *Environ Health Perspect* **2003**, *111* (4), 409-413. DOI: 10.1289/ehp.5767.
